# A Novel Reactive Oxygen Species‐Responsive Bone‐Targeted Nanoplatform Based on Cloperastine Attenuates Ovariectomy‐Induced Bone Loss by Inhibiting Osteoclast Oxidative Phosphorylation

**DOI:** 10.1002/advs.78205

**Published:** 2026-10-11

**Authors:** Haibo Liang, Yong Xiao, Jiangtao Luo, Xiao Yang, Yayun Yang, Qihang Wu, Chenxin Yu, Zhihao Zhou, Xiaoqing Si, Jiansen Miao, Yiting Tu, Qichen Lu, Boreng Qian, Xiangyang Wang, Jiake Xu, Changcan Shi, Haiming Jin

**Affiliations:** ^1^ Department of Orthopaedics Key Laboratory of Orthopaedics of Zhejiang Province The Second Affiliated Hospital and Yuying Children's Hospital of Wenzhou Medical University Wenzhou People's Republic of China; ^2^ School of the Second Clinical Medical Sciences Wenzhou Medical University Wenzhou People's Republic of China; ^3^ Wenzhou Institute University of Chinese Academy of Sciences Wenzhou People's Republic of China; ^4^ Department of Spinal Surgery School of Medicine Shanghai East Hospital Tongji University Shanghai People's Republic of China; ^5^ Department of Endocrinology The Second Affiliated Hospital and Yuying Children's Hospital of Wenzhou Medical University Wenzhou People's Republic of China; ^6^ Faculty of Pharmaceutical Sciences Shenzhen University of Advanced Technology Shenzhen People's Republic of China; ^7^ School of Biomedical Sciences The University of Western Australia Perth Australia

**Keywords:** bone target, nanomedicine, osteoclastogenesis, osteoporosis, oxidative phosphorylation

## Abstract

Postmenopausal osteoporosis is caused by excessive osteoclast‐mediated bone resorption. This study identifies mitochondrial oxidative phosphorylation (OXPHOS) as a tractable metabolic target for antiresorptive therapy. A literature‐guided drug repurposing screen focused on compounds reported to affect OXPHOS or related energy metabolism identified cloperastine (Clo) as a potent suppressor of receptor activator of nuclear factor κB ligand‐induced osteoclastogenesis. Clo reduces mitochondrial respiration, downregulates OXPHOS complex proteins, decreases mitochondrial reactive oxygen species (ROS), dissipates the mitochondrial membrane potential, disrupts mitochondrial dynamics, and collectively impairs osteoclast differentiation and bone resorption in vitro. Asp8‐modified methionine/lysine‐based ROS‐responsive polyethylene glycol micelles encapsulating Clo (Clo/Asp8‐PMK‐MCs) were developed for potential bone‐directed delivery. These micelles (∼220 nm; −3.60 ± 0.09 mV) exhibit ROS‐triggered disassembly and accelerated Clo release under oxidative conditions. Fluorescence tracking of DID‐labeled micelles shows stronger skeletal signals with Asp8 modification than without it. Clo/Asp8‐PMK‐MCs inhibit osteoclast differentiation and bone resorption, whereas bioactive Asp8‐PMK‐MCs simultaneously promote osteoblast differentiation and matrix mineralization. In ovariectomized mice, Clo/Asp8‐PMK‐MCs attenuate ovariectomy‐induced bone loss, preserve trabecular bone microarchitecture, reduce osteoclast‐related parameters and serum bone resorption markers, and enhance bone formation. These findings support Clo/Asp8‐PMK‐MCs as a potential OXPHOS‐targeted nanomedicine integrating Clo‐mediated antiresorptive activity with carrier‐mediated osteogenic activity to attenuate estrogen deficiency‐induced bone loss.

## Graphical abstract

Cloperastine suppresses osteoclastogenesis by inhibiting PGC1β‐mediated oxidative phosphorylation. Its incorporation into a ROS‐responsive, Asp8‐modified nanoplatform enhances skeletal accumulation. The resulting Clo/Asp8‐PMK‐MCs combine cloperastine‐mediated antiresorptive activity with carrier‐mediated osteogenic activity to attenuate OVX‐induced bone loss.

## 1 Introduction

Osteoporosis is a prevalent skeletal disorder characterized by reduced bone mass, trabecular microarchitecture deterioration, and an increased risk of fracture [1]. Among its subtypes, postmenopausal osteoporosis is the most common and is primarily driven by excessive osteoclast (OC)‐mediated bone resorption resulting from estrogen deficiency [2]. Although currently available antiresorptive therapies, including bisphosphonates and denosumab, effectively reduce fracture risk, their long‐term clinical use is constrained by adverse effects. These effects include atypical fractures, osteonecrosis of the jaw, and rebound bone loss following treatment discontinuation in some patients [3, 4]. Collectively, these limitations highlight the need for novel therapeutic strategies that safely, effectively, and durably suppress pathological bone resorption.

OC differentiation and bone resorption are high energy‐demanding processes, and metabolic reprogramming is indispensable for osteoclastogenesis [5, 6]. Among the metabolic pathways involved, mitochondrial oxidative phosphorylation (OXPHOS) has emerged as a central regulator of OC differentiation and resorptive activity by supplying the high levels of adenosine triphosphate (ATP) required to sustain these energy‐intensive processes [6]. Experimental inhibition of mitochondrial respiration markedly impairs OC formation and bone‐resorptive function [7]. This highlights mitochondrial metabolism as a promising therapeutic target for OC‐driven bone loss [8]. Nevertheless, therapeutic strategies that specifically suppress OC activity by targeting mitochondrial OXPHOS remain insufficiently explored.

Drug repurposing is an efficient strategy for identifying new therapeutic indications for approved drugs with well‐established safety profiles, thereby accelerating clinical translation [9, 10]. Based on this concept, we established a literature‐guided drug repurposing screen focused on compounds reported to affect mitochondrial OXPHOS or related energy metabolism, with the aim of identifying inhibitors of OC differentiation. Cloperastine (Clo), a clinically used antitussive agent, was included in the screen because previous findings associated Clo treatment with reduced abundance of proteins related to mitochondrial respiratory‐chain complexes I and IV, suggesting a potential influence on mitochondrial oxidative metabolism [11]. However, the role of Clo in bone remodeling, particularly its effects on OC metabolism and mitochondrial function, has not yet been explored. Considering the essential role of OXPHOS in supplying energy to sustain OC differentiation, we hypothesized that pharmacological disruption of mitochondrial energy metabolism could suppress OC differentiation and bone resorption, and investigated Clo as a repurposing candidate.

A major challenge in small‐molecule antiresorptive therapy is the lack of bone specificity, which often results in extensive systemic exposure and off‐target adverse effects [1, 12, 13]. Accordingly, bone‐targeted nanocarriers have attracted growing interest as strategies for enhancing drug accumulation at skeletal sites, while minimizing systemic toxicity [12, 14, 15]. Acidic aspartate oligopeptides, such as Asp8, exhibit a strong affinity for hydroxyapatite and have been widely exploited to guide nanoparticles to bone surfaces [12, 16]. In parallel, reactive oxygen species (ROS) levels are elevated in OC‐rich and osteoporotic microenvironments [17]. This provides a rationale for ROS‐responsive drug release [14]. Therefore, integrating bone‐targeting ligands with ROS‐responsive polymeric carriers offers a rationale for delivering mitochondria‐modulating small molecules to skeletal sites and promoting drug release under oxidative conditions.

In this study, we integrated literature‐guided compound screening with metabolic targeting and bone‐directed nanodelivery to investigate a therapeutic approach for OC‐driven bone loss. First, the dynamic regulation of mitochondrial OXPHOS during OC differentiation was delineated, and Clo was identified as a potent inhibitor of receptor activator of nuclear factor κB ligand (RANKL)‐induced osteoclastogenesis in association with the suppression of mitochondrial OXPHOS. Thereafter, an Asp8‐modified ROS‐responsive mPEG‐PMK micellar system was engineered to encapsulate Clo (Clo/Asp8‐PMK‐MCs) and its physicochemical properties, cytocompatibility, skeletal distribution, and cellular uptake were systematically characterized. Using in vitro assays, the effects of Clo and Clo/Asp8‐PMK‐MCs on OC differentiation, mitochondrial function and dynamics, and bone resorption, together with the effects of the micellar carrier on osteoblast (OB) differentiation and matrix mineralization were evaluated. Finally, using an ovariectomized mouse model, the effects of Clo and Clo/Asp8‐PMK‐MCs on the bone mass, trabecular microarchitecture, bone remodeling parameters, and histological features of major organs were assessed. Collectively, these results support the potential of repurposed Clo‐based, OXPHOS‐targeted bone‐directed nanomedicine to attenuate OC‐mediated bone loss.

## 2 Materials and Methods

### 2.1 Reagents and Antibodies

A Food and Drug Administration (FDA)‐approved drug library was purchased from MedChem Express (New Jersey, USA). Recombinant macrophage colony‐stimulating factor (M‐CSF) and RANKL were obtained from R&D Systems (Minneapolis, USA). Essential reagents including 4% paraformaldehyde (PFA), phosphate‐buffered saline (PBS), ethylenediaminetetraacetic acid (EDTA) decalcification solution, and Tris‐buffered saline with Tween (TBST) were purchased from Servicebio (Wuhan, China). Triton X‐100 and dimethyl sulfoxide (DMSO) were purchased from SolarBio (Beijing, China). Alpha Modification of Eagle's Minimum Essential Medium (α‐MEM) was purchased from CIENRY Biotechnology (Huzhou, China). Fetal bovine serum (FBS) and MitoSOX Red were purchased from Thermo Fisher Scientific (Waltham, MA). ROS Assay Kit, protease/phosphatase inhibitor, JC‐1 kit, and radioimmunoprecipitation assay (RIPA) buffer were acquired from Beyotime Biotechnology (Shanghai, China). Primary antibodies against cathepsin K (CTSK), nuclear factor of activated T cells 1 (NFATc1), ATP synthase F1 subunit alpha (ATP5A), and ubiquinol‐cytochrome c reductase core protein 2 (UQCRC2) were obtained from Santa Cruz Biotechnology (CA, USA). Antibodies for mitochondrially encoded cytochrome c oxidase II (MTCO2), succinate dehydrogenase complex iron sulfur subunit B (SDHB), NADH:ubiquinone oxidoreductase subunit B8 (NDUFB8), β‐actin, dynamin‐related protein 1 (DRP1), mitofusin 2 (MFN2), optic atrophy protein 1 (OPA1), peroxisome proliferator‐activated receptor gamma coactivator 1‐beta (PGC1β), integrin β3, hexokinase 2 (HK2), pyruvate kinase M2 (PKM2), lactate dehydrogenase A (LDHA), collagen type I alpha 1 chain (COL1A1), Runt‐related transcription factor 2 (RUNX2), and osteocalcin (OCN) were obtained from Proteintech Group (Wuhan, China). The c‐Fos antibody was purchased from Abcam (Cambridge, USA). Cell culture reagents were purchased from Gibco (Carlsbad, CA, USA). MAL‐mPEG5K‐NH2, methionine monomer, and Nε‐trifluoroacetyl‐L‐lysine were purchased from WEIHUA BIO (Guangzhou, China). Asp8 was purchased from QYAOBIO (Shanghai, China), and N, N‐dimethylformamide (DMF) was purchased from Aladdin (Shanghai, China). Tetrahydrofuran (THF) was purchased from Energy Chemicals (Anhui, China). A list of all abbreviations used in this manuscript is provided in Table S1. Detailed information for all antibodies, including the supplier, catalog number, and dilution, is provided in Table S2.

### 2.2 Bioinformatic Analysis

The public bulk RNA‐seq dataset GSE225974 was obtained from GEO and analyzed separately. For both datasets, differentially expressed genes were identified using p < 0.05 and |log_2_ fold change| > 1. Differentially expressed genes were subjected to Gene Ontology biological process (GO‐BP) and Kyoto Encyclopedia of Genes and Genomes (KEGG) enrichment analyses. Gene set enrichment analysis (GSEA) was performed using ranked gene lists. PGC‐1β expression across cell types was retrieved directly from the Human Protein Atlas (https://www.proteinatlas.org/), without additional computational analysis.

Single‐cell RNA‐sequencing data from GSE147174 were obtained from the GEO database. Empty droplets were removed using emptyDrops (FDR ≤ 0.01), followed by doublet removal with scDblFinder and ambient RNA correction with DecontX. Low‐quality cells were excluded based on outlying UMI counts, detected gene numbers, and mitochondrial transcript proportions using a 3‐MAD criterion. The processed data were analyzed using Seurat for normalization, PCA, clustering, and UMAP visualization. Cell types were annotated according to differentially expressed genes and canonical markers. Hallmark pathway enrichment was assessed by GSVA, and oxidative phosphorylation activity was evaluated using Hallmark and KEGG pathway gene sets.

### 2.3 Literature‐Guided Compound Selection and Screening

A literature‐guided drug repurposing screen was conducted to identify compounds reported to affect mitochondrial OXPHOS or related energy metabolism for evaluation in OC differentiation assays. PubMed was searched in May 2023 using the following query: FDA[Title/Abstract] AND (respiratory chain[Title/Abstract] OR electron transport chain[Title/Abstract] OR oxidative respiratory chain[Title/Abstract] OR oxidative phosphorylation[Title/Abstract] OR mitochondrial complex[Title/Abstract] OR ATP synthase[Title/Abstract])

All retrieved records were screened by title and abstract, with full‐text assessment when necessary to determine relevance. Initial candidate identification was exploratory, without prespecified formal inclusion or exclusion criteria, and was guided by published reports of effects on OXPHOS or related energy metabolism. This process yielded 43 candidate compounds. OXPHOS‐related annotations and FDA‐approval information were obtained from the cited publications. The literature‐reported effects and supporting references for each compound are provided in Table S3. Selected compounds were obtained from the MCE FDA‐approved drug library (HY‐LD‐000001444) when available and purchased individually otherwise.

### 2.4 Mitochondrial Stress Test

Mitochondrial respiration was evaluated by measuring the oxygen consumption rate (OCR) using a Seahorse XF extracellular flux analyzer (Agilent) and a Mito Stress Test kit, following the manufacturer's instructions. Briefly, 2 × 10^4^ bone marrow‐derived macrophages (BMM) were seeded per well in XFe96 plates, treated as indicated, and induced with RANKL for five days to generate OCs. One hour before the assay, cells were equilibrated at 37°C in a non‐CO_2_ incubator with assay medium (Seahorse XF base medium supplemented with 1 mm sodium pyruvate, 2 mm L‐glutamine, and 10 mm D‐glucose). Mitochondrial modulators were injected sequentially at optimized final concentrations: oligomycin (1.5 µm; ATP synthase inhibitor), FCCP (3 µm; uncoupler), and rotenone/antimycin A (0.5 µm each; Complex I/III inhibitors). OCR values were normalized to the total protein content.

### 2.5 Cell Cultures

BMMs were isolated from the femurs of six‐week‐old C57BL/6 mice and expanded in α‐MEM containing 10% FBS, 1% penicillin–streptomycin (P/S), and 50 ng/mL M‐CSF at 37°C in 5% CO_2_. The first‐passage cells were then used for assays. For OC induction, the cells were seeded at 1 × 10^4^ per well in 96‐well plates and stimulated with RANKL (50 ng/mL). Primary OBs were obtained from neonatal mouse calvariae via sequential digestion with 0.25% trypsin and 0.2% collagenase II. The cells were then maintained in α‐MEM supplemented with 10% FBS and 1% P/S. The early‐passage (≤P2) OBs were used in all experiments.

### 2.6 Western Blotting

After treatment, the cells were rinsed with PBS and lysed on ice in RIPA buffer supplemented with protease and phosphatase inhibitors for 30 min. Lysates were collected by scraping and clarified. The protein content was then quantified using a bicinchoninic acid kit, and samples were normalized to equal concentrations. Equal amounts of proteins were separated on 12% sodium dodecyl sulfate–polyacrylamide gel electrophoresis gels and transferred onto polyvinylidene fluoride membranes. Membranes were blocked with 5% skim milk in TBST for 2 h and subsequently incubated with primary antibodies at 4°C overnight. After TBST washes, membranes were incubated with horseradish peroxidase‐conjugated secondary antibodies for 2 h. Signals were developed using enhanced chemiluminescence reagent (Meilunbio) and imaged on a ChemiDoc XRS+ system. The band intensities were quantified using ImageJ.

### 2.7 Tartrate‐Resistant Acid Phosphatase Staining

The BMMs were seeded at 1 × 10^4^ per well in 96‐well plates. The following day, the medium was replaced with complete medium containing RANKL (50 ng/mL) and the indicated interventions and refreshed every two days until day 6 to allow OC maturation. For TRAP staining, cells were fixed with 4% PFA for 15 min. The tartrate‐resistant acid phosphatase (TRAP) working solution was prepared according to the manufacturer's instructions (Sigma–Aldrich, Australia), added at 50 µL per well, and the plates were incubated at 37°C for 1 h before three PBS washes.

### 2.8 Cell Viability Assay

BMMs were seeded at 1 × 10^4^ cells per well, and OBs were seeded at 5 × 10^3^ cells per well in 96‐well plates. These cells were cultured under the indicated conditions for four days. On day 4, viability was assessed using Cell Counting Kit (CCK)‐8 (Vazyme, Nanjing, China) according to the manufacturer's instructions. The plates were incubated with reagent for 2 h at 37°C, and absorbance was read at 450 nm on a microplate reader (Thermo, Rockford, USA).

BMM cytotoxicity was also evaluated using an lactate dehydrogenase (LDH) Cytotoxicity Assay Kit (MA0649; Meilunbio, Dalian, China). BMMs were seeded in 96‐well plates and treated with 0–20 µm Clo for 48 or 96 h. LDH release was measured according to the manufacturer's instructions, and absorbance was recorded at 490 nm.

### 2.9 F‐Actin Staining

BMMs were seeded at 1 × 10^4^ cells per well in 96‐well plates, and osteoclastogenesis was induced. The following day, the medium was replaced with complete medium containing RANKL (50 ng/mL) and the indicated interventions. After four days, F‐actin was stained using a rhodamine–phalloidin kit (Beyotime, China), and the nuclei were counterstained with 4′,6‐diamidino‐2‐phenylindole (DAPI). Multinucleated cells bearing ≥3 nuclei were defined as OCs, and the number of nuclei per OC was quantified.

### 2.10 Immunofluorescence

BMMs were seeded at 1 × 10^5^ cells per 35‐mm glass‐bottom confocal dish, stimulated with RANKL for five days, and treated as indicated. Cells were fixed with 4% PFA for 15 min, permeabilized with Triton X‐100 for 5 min, and blocked with 10% goat serum for 30 min at 37°C. Primary antibodies against NFATc1 and CTSK (1:50 in PBS) were applied overnight at 4°C. After equilibration for 1 h at 37°C and PBS washes, cells were incubated with appropriate fluorescent secondary antibodies (Proteintech, 1:200) for 1 h at 37°C. Nuclei were counterstained with DAPI, and images were acquired using a fluorescence microscope (Keyence, Japan).

Bone sections were subjected to antigen retrieval by pepsin digestion (ZSGB‐BIO) at 37°C for 1 h. After washing with PBS, sections were blocked with 10% goat serum at 37°C for 1 h and incubated with primary antibodies against CTSK (1:50) and PGC1β (1:100) overnight at 4°C. Following PBS washes, sections were incubated with the corresponding fluorescent secondary antibodies (Proteintech, 1:200) at 37°C for 1 h in the dark. Sections were then mounted using a DAPI‐containing mounting medium. Images were acquired using a fluorescence microscope (Keyence, Japan). PGC1β, CTSK, and nuclei were visualized in green, red, and blue, respectively. Corresponding channels were merged to examine the spatial distribution of PGC1β and CTSK signals in bone tissue.

### 2.11 RNA Sequencing

BMMs were seeded in six‐well plates at 4 × 10^5^ cells per well and induced with RANKL for five days. The cells were washed thrice with ice‐cold PBS, lysed on ice with TRIzol reagent (Yeasen, China) for 5 min, and the lysates were transferred to RNase‐free microtubes. Samples were snap‐frozen on dry ice and shipped to Personalbio (Wuhan, China) for transcriptome (RNA‐sequencing [RNA‐seq]) library preparation and sequencing. The sequencing data generated in this study have been submitted to the Sequence Read Archive under BioProject accession number PRJNA1531789.

### 2.12 Measurement of Mitochondrial Complex I Activity

BMMs were cultured in 100‐mm dishes with or without RANKL and treated with vehicle or 10 µm Clo for 5 days. Mitochondria were isolated using the Mitochondria Isolation Kit for Cultured Cells (ab110170, Abcam). Complex I activity was measured using the Complex I Enzyme Activity Microplate Assay Kit (ab109721, Abcam) according to the manufacturer's instructions, with 20 µg of mitochondrial protein loaded per well. Absorbance at 450 nm was recorded kinetically, and activity was calculated from the background‐corrected linear reaction rate and expressed as mOD/min.

### 2.13 ROS Level Detection

BMMs were cultured for five days under the indicated conditions in 35‐mm dishes. ROS levels were assessed according to the manufacturer's protocol (Beyotime). Briefly, cells were incubated with 10 µm 2′,7′‐dichlorodihydrofluorescein diacetate in serum‐free α‐MEM at 37°C for 30 min in the dark. The cells were then washed thrice with serum‐free medium. Fluorescence images (excitation at 488 nm) were acquired using a fluorescence microscope, and signal intensities were quantified using ImageJ software.

### 2.14 Mitochondrial ROS Detection

Mitochondrial superoxide levels were assessed using the MitoSOX Red reagent (Thermo Fisher Scientific). After treatment, cells were incubated in serum‐free medium containing 1 µm MitoSOX Red at 37°C for 30 min in the dark. The cells were then washed thrice with PBS. Fluorescence images were acquired using fluorescence microscopy, and the signal intensity was quantified using ImageJ.

### 2.15 Mitochondrial Membrane Potential

The mitochondrial membrane potential (ΔΨm) was assessed using the JC‐1 assay. The working solution was prepared by mixing the JC‐1 dye with JC‐1 buffer (1:200). Treated cells were incubated with the working solution at 37°C for 20 min, washed twice with JC‐1 buffer, and replenished with fresh culture medium. Fluorescence images were captured using a fluorescence microscope. Membrane potential was quantified as the red/green fluorescence ratio (J‐aggregates/J‐monomers), where a lower ratio indicated mitochondrial depolarization (reduced ΔΨm) and a higher ratio reflected hyperpolarization (elevated ΔΨm).

### 2.16 Synthesis of Carrier Components

#### 2.16.1 Synthesis of mPEG_5K_‐PMK

Amine‐terminated methoxy poly(ethylene glycol) (mPEG_5K_‐NH2; 0.300 g, approximately 0.060 mmol, 1.0 equiv), L‐methionine N‐carboxyanhydride (L‐Met NCA; 0.189 g, 1.08 mmol, 18 equiv), and Nε‐trifluoroacetyl‐L‐lysine N‐carboxyanhydride [Nε‐TFA‐L‐Lys NCA; 0.483 g, 1.80 mmol, 30 equiv] were dissolved in anhydrous N,N‐dimethylformamide (20 mL). The reaction mixture was stirred under a nitrogen atmosphere at 40°C for 72 h.

#### 2.16.2 Synthesis of MAL‐mPEG_5K_‐PMK

Maleimide‐functionalized poly(ethylene glycol) amine (MAL‐PEG_5K_‐NH_2_; 0.300 g), L‐methionine N‐carboxyanhydride (L‐Met NCA; 0.189 g), and Nε‐trifluoroacetyl‐L‐lysine N‐carboxyanhydride [Nε‐TFA‐L‐Lys NCA; 0.483 g] were dissolved in anhydrous N,N‐dimethylformamide (DMF; 20 mL). The reaction mixture was stirred under a nitrogen atmosphere at 40°C for 72 h. After completion of the reaction, the mixture was concentrated under reduced pressure and dialyzed against deionized water for 24 h. The dialysate was lyophilized, and the resulting material was redispersed in DMF (4 mL). The solution was added dropwise to ice‐cold diethyl ether (30 mL) to precipitate the product. The precipitate was collected and dried under vacuum to obtain MAL‐PEG_5K_‐PMK.

#### 2.16.3 Synthesis of Asp8‐mPEG_5K_‐PMK

To obtain the target polymer (Asp8‐mPEG_5K_‐PMK), the maleimide group of PMK‐mPEG_5K_‐MAL was coupled to a D‐Asp8 peptide bearing a C‐terminal thiol. Asp8 (10 mg) was dissolved in DMF (20 mL) containing PMK‐mPEG5K‐MAL (200 mg), and the mixture was stirred overnight at room temperature. The crude product was dialyzed for 12 h to remove excess peptides and lyophilized to yield PMK‐mPEG5K‐Asp8 as a white solid.

#### 2.16.4 Characterization and ROS‐Responsive Degradation

PMK‐mPEG‐MAL, PMK‐mPEG, and PMK‐mPEG‐Asp8 (25 mg/mL) were characterized using ^1^H nuclear magnetic resonance (NMR) spectroscopy in DMSO‐d6. To evaluate PMK copolymer degradation under simulated ROS conditions, PMK‐mPEG_5K_‐Asp8 was dissolved in D_2_O containing various concentrations of H_2_O_2_ and incubated for 24 h. The ^1^H NMR spectra were then recorded to monitor the change in the integral ratio of the thioether signal (δ ≈ 2.15 ppm) to the sulfoxide signal (δ ≈ 2.79 ppm).

### 2.17 Preparation of ROS‐Responsive Clo‐Loaded PMK‐Micelles (MCs)

#### 2.17.1 Formulation of Targeted PMK‐MCs

PMK‐mPEG (50 mg), PMK‐mPEG_5K_‐Asp8 (5 mg; mass ratio PMK‐PEG5K‐OCH_3_: PMK‐mPEG_5K_‐Asp8 ≈ 10:1), and Clo (5 mg) were co‐dissolved in THF (6 mL). The organic solution was slowly injected via a syringe into vigorously stirred deionized water (10 mL) to obtain a uniform suspension (nanoprecipitate). To remove THF and adjust the final volume to 10 mL, the suspension was incubated in a 37°C vacuum bath.

#### 2.17.2 Formulation of Non‐Targeted PMK‐MCs

For non‐targeted control, PMK‐mPEG_5K_‐Asp8 was replaced with PMK‐mPEG_5K_ while keeping all other steps identical.

### 2.18 Characterization of ROS‐Responsive Clo‐Loaded Asp8‐PMK‐MCs

The hydrodynamic diameter, polydispersity, and zeta potential were quantified using dynamic light scattering (DLS), and the particle morphology was visualized using scanning electron microscopy (SEM). ROS responsiveness was evaluated by tracking the size evolution of blank Asp8‐PMK‐MCs dispersed in distilled water with or without 10 mM H_2_O_2_, measured at 6 and 24 h via DLS (Zetasizer Nano ZS, ZEN3600; Malvern, UK). Morphological alterations of Clo/Asp8‐PMK‐MCs after incubation in 10 mM H_2_O_2_ for 6 and 24 h were examined via SEM operated at 5 kV.

### 2.19 In Vitro ROS‐Triggered Release of Clo From Asp8‐PMK‐MCs

Clo‐loaded Asp8‐PMK‐MCs (drug equivalent: 2.0 mg Clo) were suspended in PBS (pH 7.4) containing 0.5% (v/v) Tween‐80 and incubated at 37°C in the presence of 0, 0.1 and 10 mm H_2_O_2_ to assess oxidative stress‐responsive release over 0–80 h [18]. At predefined time points, 1 mL of the dispersion was withdrawn for analysis and immediately replaced with an equal volume of fresh release medium to maintain the sink conditions. The Clo concentrations were quantified using fluorescence spectroscopy (FluoroMax‐4; excitation 265 nm, emission 341 nm) [19].

### 2.20 Calcein Acetoxymethyl Ester/Propidium Iodide Staining Assay

Under the indicated conditions, cells were incubated with a working solution of Calcein acetoxymethyl ester (AM) and propidium iodide (PI) at 1:1000 in serum‐free medium for 30 min at 37°C in the dark. Fluorescence images were acquired using a Keyence fluorescence microscope. Calcein AM positivity indicated live cells, whereas PI positivity indicated dead cells. The percentage of dead cells (PI‐positive/total) was quantified using ImageJ.

### 2.21 In Vivo Bone‐Targeting of DID‐Labeled Asp8‐PMK‐MCs

To evaluate the bone‐targeting capability of Asp8‐PMK‐MCs in vivo, mice received an intravenous injection (100 µL) of DID‐labeled PMK‐MCs with or without Asp8 modification (DID, 20 µg mL^−^^1^) [20]. Six hours after injection, the mice were anesthetized and imaged using an in vivo fluorescence imaging system (Newton 7.0, France), followed by euthanasia. Tissues were collected for ex vivo fluorescence analysis to determine the biodistribution of DID‐labeled PMK‐MCs.

### 2.22 Cellular Uptake of DID‐Loaded PMK‐MCs in BMMs and OBs

DID (Meilun Bio, China) was used as a fluorescent probe to label PMK‐MCs [20]. DID‐loaded polymeric micelles were prepared by following the same procedure used for Clo‐loaded micelles. BMMs or OBs were seeded on 35‐mm confocal dishes. After attachment, the culture medium was replaced with one of the following in α‐MEM containing 2% FBS: free DID, DID/PMK‐MCs, or DID/Asp8‐PMK‐MCs. After cells were incubated at 37°C for 1, 3, or 6 h, the DID‐containing medium was replaced with fresh α‐MEM, and cells were washed thrice with PBS. Samples were then fixed with 4% PFA for 10 min and counterstained with DAPI for 10 min. Cellular fluorescence was directly visualized using a fluorescence microscope (Keyence, Japan).

### 2.23 Bone Resorption Assay

Bone slices prepared from bovine tibiae (FuChi Medical Technology Studio, Shanghai, China) were sterilized in 75% ethanol and soaked overnight in α‐MEM containing 1% P/S. The next day, the slices were placed in 96‐well plates, and BMMs were seeded onto each slice at 1 × 10^4^ cells per well. Cells were cultured in a medium containing RANKL until mature OCs were formed. Thereafter, the indicated interventions were applied for two days to assess their effects on bone resorption. The cells were then removed from the slice surfaces via trypsinization, the slices were air‐dried, and the resorption pits were imaged using SEM. The resorbed areas were quantified using QuPath software (v0.6.0).

### 2.24 Real‐Time Quantitative Polymerase Chain Reaction (RT‐qPCR)

Total RNA was extracted using TRIzol reagent and reverse‐transcribed into complementary DNA using a reverse transcription kit (Thermo Fisher Scientific) according to the manufacturer's instructions. Quantitative polymerase chain reaction (qPCR) was performed with gene‐specific primers under the following cycling conditions: initial denaturation at 94°C for 5 min, followed by 30 cycles of denaturation at 94°C for 40 s, annealing at 60°C for 40 s, and extension at 72°C for 40 s, with a final extension at 72°C for 5 min. Relative gene expression was quantified using the 2^−ΔΔCt^ method, with β‐actin serving as the internal reference gene. The primer sequences used in this study are listed in Table S4.

### 2.25 Molecular Docking

Two‐dimensional (2D) structures of small‐molecule ligands were retrieved from PubChem (pubchem.ncbi.nlm.nih.gov), converted to three‐dimensional (3D) conformations in ChemOffice, and saved as MOL2 files. The available crystal structure of the peroxisome proliferator‐activated receptor (PPAR)–PGC1β complex (PDB ID: 3SP6) was obtained from the Research Collaboratory for Structural Bioinformatics Protein Data Bank and used for molecular docking. The structures were preprocessed in PyMOL by removing crystallographic water and non‐essential heteroatoms/ions, such as phosphate. The structures were then saved as PDB files. Docking was performed using AutoDock Vina software. Protein and ligand structures were prepared using AutoDockTools (v1.5.6). Hydrogen was added, water was removed from proteins, ligand torsions were defined, and charges were assigned. The docking search space (grid box) was centered on known or predicted binding sites. Poses were ranked according to Vina binding affinity (kcal/mol), and the top‐scoring conformation was used as the predicted binding mode. Ligand–receptor interactions were visualized and analyzed using Discovery Studio 2019 to generate 2D interaction maps and 3D contact views of key residues. The docking analysis was used to predict the potential interaction mode between Clo and the PPARα–PGC1β complex and was interpreted as supportive computational evidence rather than definitive proof of direct physical binding.

### 2.26 Cellular Thermal Shift Assay (CETSA)

The BMMs underwent freeze‐thaw cycles thrice using liquid nitrogen and a 37°C water bath. The cell suspension was then evenly divided into two tubes. One tube received Clo with a final concentration of 10 µm, whereas the other received an equal volume of PBS as a control. This was followed by incubation on ice for 1 h. The lysates from each group were further divided into eight equal portions, each subjected to heat treatment at temperatures of 37°C–65°C for 3 min. Subsequently, cell lysates were centrifuged, and the supernatant proteins were mixed with protein loading buffer before undergoing western blot analysis to assess PGC1β levels [21].

### 2.27 Lentiviral Transduction and siRNA Transfection

Lentiviral vectors for mouse PGC1β overexpression (NCBI RefSeq: NM_133249.3) were purchased from Applied Biological Materials (ABM, Richmond, BC, Canada). The viral titer was 1 × 10^8^ TU/mL, and cells were transduced at a multiplicity of infection (MOI) of 40. Small interfering RNAs (siRNAs) were purchased from GenePharma (Shanghai, China), and their sequences are listed in Table S5. Cells were transfected with siRNAs at a final concentration of 50 nM using CALNP RNAi transfection reagent (DuoNa, Beijing, China) according to the manufacturer's instructions. Following lentiviral transduction or siRNA transfection, mRNA and protein levels were assessed at 24 and 72 h, respectively.

### 2.28 OB Differentiation

Osteogenic differentiation was induced in α‐MEM supplemented with 10% FBS, 1% P/S, dexamethasone (10 nm), ascorbic acid (50 µg/mL), and β‐glycerophosphate (10 mm). During induction, the indicated interventions were administered as specified. Alkaline phosphatase (ALP) staining (day 7) and Alizarin Red staining (ARS) (day 21) were performed according to the manufacturer's instructions (Beyotime, China) [22].

### 2.29 Animals

All animal procedures complied with the guidelines of the Wenzhou Institute, University of Chinese Academy of Sciences (Ethics No. WIUCAS25022008). Twelve‐week‐old female C57BL/6 mice were obtained from the Wenzhou Institute, acclimated for one week, and anesthetized with isoflurane prior to sham operation or bilateral ovariectomy (OVX) [14]. Animals were randomized into the following nine groups (n = 6 each): for the drug study—Sham, OVX, Clo low dose (5 mg/kg), and Clo high dose (10 mg/kg); for the materials study—Sham, OVX, Clo, Asp8‐PMK‐MCs, and Clo/Asp8‐PMK‐MCs. Beginning seven days after surgery, the Sham and OVX + vehicle groups received saline, the Clo group received Clo (dissolved in saline) via tail vein injection, and the Asp8‐PMK‐MCs and Clo/Asp8‐PMK‐MCs groups received tail vein injections of the corresponding materials with equivalent amounts of Clo. All treatments were administered every other day for six weeks, with saline volumes matched across groups. The mice were monitored throughout the experiment and were maintained in good condition. At the endpoint, the animals were euthanized via cervical dislocation under isoflurane anesthesia, and the femora were collected for histology and micro‐computed tomography (micro‐CT). To assess in vivo bone formation, calcein (25 mg/kg; Solarbio, Beijing, China) was intravenously injected seven and three days before euthanasia to generate double labels [23]. Interlabel distances were measured in ImageJ and divided by the four‐day interval to calculate the mineral apposition rate (MAR).

An additional cohort of mice without OVX was randomized into four groups (n = 6 per group): Control, free Clo, Asp8‐PMK‐MCs, and Clo/Asp8‐PMK‐MCs. Treatments were administered via tail vein injection every other day for six weeks at the same doses used in the materials study. At the endpoint, blood samples were collected for complete blood counts and biochemical analysis using a BC‐5000 Vet hematology analyzer and a BS‐240 Vet chemistry analyzer (Mindray Animal), respectively. Major organs (heart, liver, spleen, lungs, and kidneys) were harvested, embedded, sectioned, and stained with H&E for histopathological evaluation.

Animals were randomly allocated to the experimental groups, with six mice per group in both the efficacy and biosafety studies. Investigators performing outcome assessments were blinded to group allocation. No exclusion criteria were prespecified. No animals died or were excluded during the experiments, and all animals were included in the analyses. The sample size was selected based on previous studies using comparable animal models and outcome measures [14].

### 2.30 Histology and Micro‐CT

The femora were fixed in 4% PFA for 48 h after harvesting. Micro‐CT imaging was performed on a SkyScan system equipped with a 0.5‐mm aluminum filter (to reduce beam hardening) using the following settings: voxel size 9 µm, rotation step 0.4°, source current 500 µA, and tube voltage 50 kV [28]. Trabecular and cortical parameters were quantified within a region of interest that was 0.5–1.5 mm distal to the growth plate using CTAn, and 3D reconstructions were generated using CTVox. The microarchitectural indices included bone volume fraction (BV/TV), trabecular thickness (Tb.Th), trabecular number (Tb.N), and trabecular separation (Tb.Sp). Following micro‐CT, femora were decalcified in 10% EDTA (pH 7.4) for 14 days, dehydrated, paraffin‐embedded, and sectioned at 5 µm for histology. The sections were subjected to hematoxylin–eosin (H&E) and TRAP staining. Immunohistochemistry for PGC1β and RUNX2 was performed according to standard protocols to assess protein expression in bone tissue [24]. DHE staining was performed according to a previously described method [25]. Von Kossa staining was performed on undecalcified bone sections using a commercial staining kit (G1043, Servicebio). Following deplasticization and rehydration, sections were rinsed five times with ultrapure water and incubated with Von Kossa solution under ultraviolet illumination for 5–20 min. After washing with distilled water, sections were counterstained with hematoxylin for 3–5 min, differentiated, blued, and stained with eosin for 5 min. Sections were then dehydrated, cleared, and mounted with neutral resin. Calcium salt deposits appeared black or brownish‐black.

### 2.31 Analysis of Serum Biomarkers of Bone Resorption

Serum was isolated via centrifugation of whole blood at 1000 × g for 10 min at room temperature. Bone resorption markers (tartrate‐resistant acid phosphatase 5b [TRAP‐5b] and β‐C‐telopeptide of type I collagen [β‐CTx]) were quantified using enzyme‐linked immunosorbent assay kits (Elabscience, Wuhan, China) following the manufacturer's instructions.

### 2.32 Statistical Analysis

Quantitative data are presented as the mean ± standard deviation (SD), and statistical analyses were performed using GraphPad Prism version 9.5.1 (GraphPad Software, San Diego, CA, USA). Where normalization was required, relative quantitative values were normalized to the corresponding control group. No data points were excluded from the statistical analyses. Data normality was assessed using the Shapiro–Wilk test. Comparisons between two independent groups were performed using an unpaired two‐tailed Student's t‐test. Comparisons involving more than two groups with one independent variable were performed using one‐way analysis of variance (ANOVA) followed by Tukey's multiple‐comparisons test, whereas datasets involving two independent variables were analyzed using two‐way ANOVA followed by Šídák's multiple‐comparisons test. The statistical test, sample size (n), and definition of biological replicates for each dataset are specified in the corresponding figure legend. All statistical tests were two‐sided, with a significance level of α = 0.05. Error bars represent SD. Statistical significance is denoted as follows: ns, not significant; ^*^ *p* < 0.05; and ^**^ *p* < 0.01.

## 3 Results and Discussion

### 3.1 OXPHOS Increases During OC Differentiation

Mitochondrial OXPHOS provides critical energy for OC differentiation and plays an essential role in osteoclastogenesis [26]. To investigate metabolic reprogramming during OC differentiation, a publicly available single‐cell RNA sequencing dataset was analyzed (GSE147174) [27]. Three distinct cell populations were identified: monocytes (Mono), pre‐OCs, and mature OCs (Figure 1A,B). Gene set variation analysis based on hallmark pathways revealed a marked enrichment of OXPHOS in OCs compared with that in Mono and pre‐OC populations (Figure 1C). OXPHOS pathway scores progressively increased along the differentiation trajectory and were the highest in mature OCs (Figure 1D), which was further confirmed by their spatial distribution on the UMAP embedding (Figure 1E).

**FIGURE 1:**
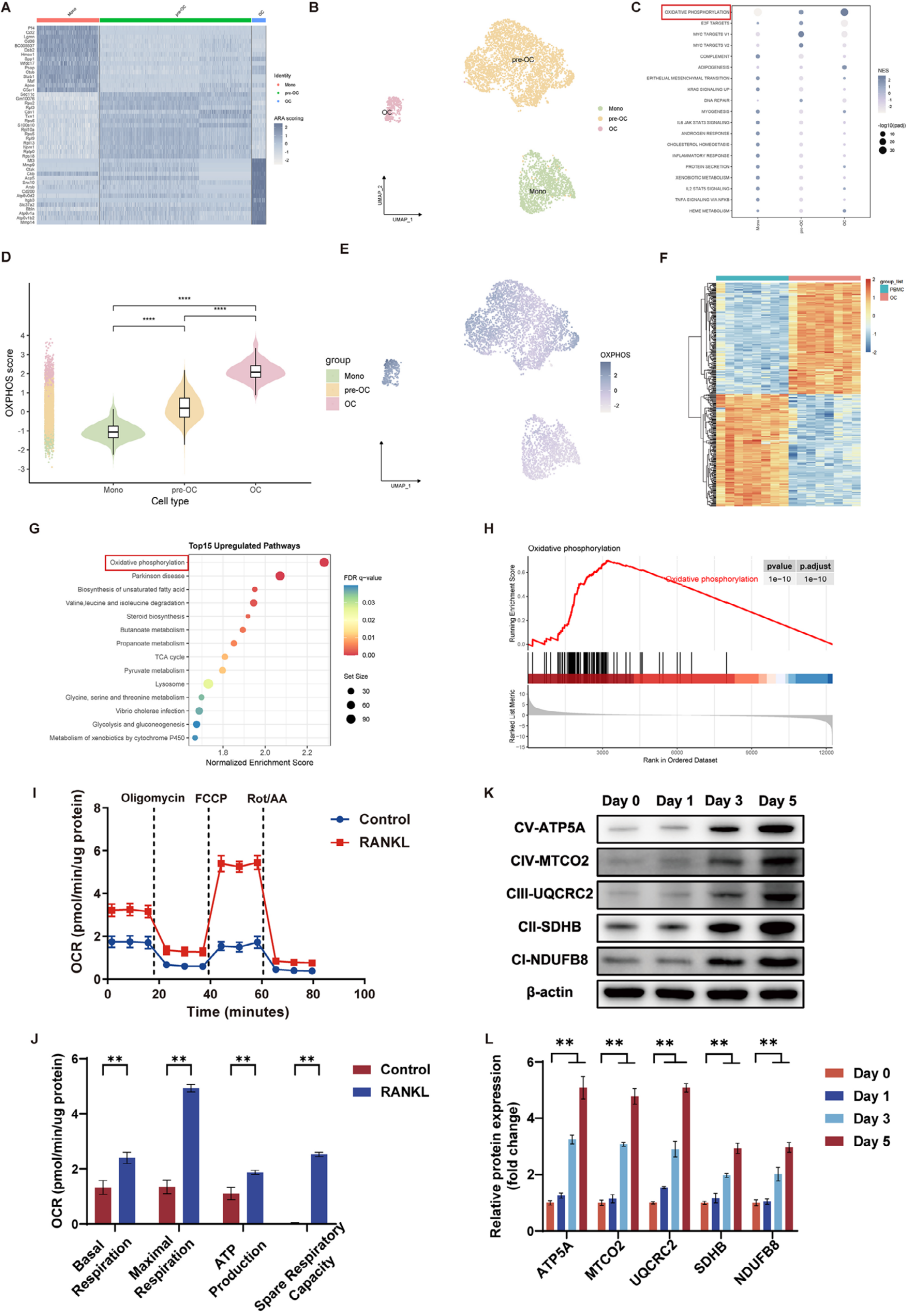
OXPHOS is upregulated during osteoclast differentiation. (A) Differential gene expression among distinct cell subpopulations during osteoclast differentiation, including Mono, pre‐OC, and OC. (B) UMAP dimensional reduction plot showing clear transcriptional clustering of the three cell populations. (C) Bubble plot of Hallmark pathway enrichment based on GSVA, demonstrating significant enrichment of OXPHOS in OCs. (D) Violin plots comparing OXPHOS pathway scores among different cell types. (E) Distribution of OXPHOS pathway scores across the UMAP embedding. (F) Heatmap showing differentially expressed genes between human PBMCs and OCs. (G) GSEA of differentially expressed genes in human PBMCs and OCs, highlighting the top 15 upregulated pathways. (H) Enrichment analysis of differentially expressed genes in human PBMCs and OCs reveals increased oxidative phosphorylation following osteoclast differentiation. (I, J) Seahorse XF96 mitochondrial stress test shows an increase in OXPHOS after OC differentiation, along with quantitative analysis of cellular respiratory parameters: basal respiration, maximal respiration, ATP production, and spare respiratory capacity. (K, L) Representative Western blots and quantification of mitochondrial OXPHOS complex proteins after 5 days of RANKL stimulation. For panels I–L, n = 3 biological replicates, each derived from BMMs independently isolated from a different mouse. Data are presented as the mean ± S.D and p values were determined using unpaired two‐tailed Student's t‐test (J), two‐way ANOVA followed by Šídák's multiple‐comparisons test (L). ns, not significant; ^*^ *p* < 0.05; ^**^ *p* < 0.01.

Consistent with single‐cell analysis, bulk RNA‐seq (GSE225974) [28] comparison between human peripheral blood mononuclear cells and OCs demonstrated distinct metabolic gene expression profiles, with OXPHOS being among the most upregulated pathways in OCs (Figure 1F–H). To functionally validate these transcriptomic findings, Seahorse mitochondrial stress tests were performed. The tests revealed that multiple mitochondrial respiratory parameters, including basal respiration, maximal respiration, ATP production, and spare respiratory capacity, were substantially increased in RANKL‐stimulated mature OCs (Figure 1I,J). In parallel, during the differentiation time course from BMMs to OCs (days 0– 5), representative proteins of mitochondrial electron transport chain complexes *I–V* showed a progressive increase (Figure 1K,L). This indicated sustained activation of electron transport chain function throughout osteoclastogenesis. Although previous studies have reported elevated OXPHOS in mature OCs [29], based on available studies, the current data provide the first time‐resolved evidence showing a gradual increase in electron transport chain complex proteins during OC differentiation (days 0–5). This further emphasizes the pivotal role of OXPHOS in OC formation.

### 3.2 Identification of Cloperastine Through a Literature‐Guided Compound Screen

Drug repurposing is an efficient approach for identifying new therapeutic indications by drawing on existing pharmacokinetic and safety data, potentially accelerating translational development [30]. Given the pivotal role of OXPHOS in OC differentiation, this study aimed to identify compounds capable of suppressing osteoclastogenesis by modulating mitochondrial oxidative metabolism. Accordingly, a literature‐guided drug repurposing search was conducted, yielding a panel of 43 candidate compounds reported to affect OXPHOS or related energy metabolism. These candidates were evaluated using a RANKL‐induced OC differentiation model.

In the primary screening, all 43 compounds were tested at a uniform concentration of 10 µm. Compounds exhibiting pronounced cytotoxicity at this dose were assessed at lower concentrations during the secondary screening. Seventeen compounds markedly suppressed OC differentiation (Figure 2A; Figure S1). To prioritize candidates with less explored roles in OC biology, compounds with prior reports in OC‐related studies were excluded from further prioritization. This refinement yielded six compounds (Clo, bedaquiline, butoconazole, lomerizine, enasidenib, and cyclobenzaprine) for which no prior OC‐related studies were identified in our literature assessment. CCK‐8 assays showed that none of the six candidates exhibited apparent cytotoxicity toward BMMs at concentrations up to 10 µm (Figure S2A). To further evaluate their effects on mitochondrial oxidative metabolism, secondary functional screening was performed using the Seahorse XF Mito Stress Test and JC‐1 staining. Among the six candidates, Clo induced the most pronounced reduction in OCR, mitochondrial ATP production, basal and maximal respiration, and spare respiratory capacity, while causing no marked alteration in extracellular acidification rate (ECAR) (Figure S2B–E). JC‐1 staining further demonstrated that all six candidates exhibited a reduction in mitochondrial membrane potential to varying degrees, indicating their potential effects on mitochondrial function (Figure S2F,G). Together, these findings support an effect of Clo on mitochondrial oxidative metabolism under the tested conditions, although they do not establish a direct molecular target within the OXPHOS machinery.Considering the overall inhibitory profile on mitochondrial oxidative metabolism, Clo was selected for subsequent mechanistic and therapeutic investigations.

**FIGURE 2:**
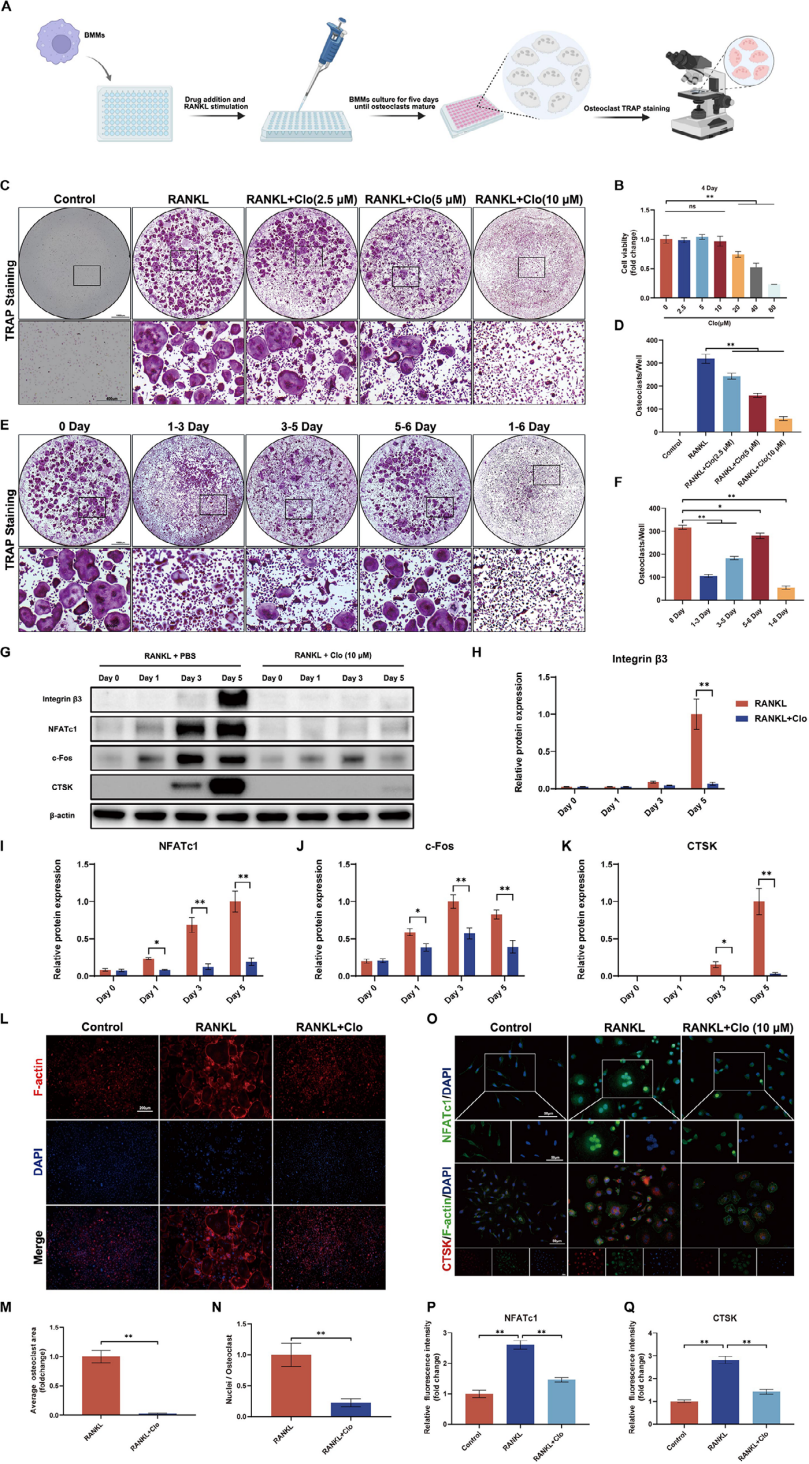
Screening of the drug Clo reveals its inhibitory effect on OC differentiation and function in vitro. (A) Schematic diagram of the drug‐screening workflow. (B) CCK‐8 assay assessing the cytotoxicity of different concentrations of Clo (0–80 µm) on BMMs. (C, D) Representative images of OC differentiation and quantification of TRAP‐positive cells after incubation with different concentrations of Clo. (E, F) Representative images of OC differentiation and quantification of TRAP‐positive cells after treatment with Clo (10 µm) at different time points. (G–K) Representative Western blots and quantitative analysis of integrin β3, CTSK, NFATc1, and c‐Fos in cells treated with Clo (10 µm) for 0, 1, 3, and 5 days. (L–N) F‐actin belt staining of OC treated with or without RANKL and Clo, and quantitative analysis of OC area and nuclei number per OC. F‐actin is shown in red, and DAPI‐stained nuclei are shown in blue. (O–Q) Immunofluorescence images and quantitative analysis of NFATc1 and CTSK in OC treated with or without RANKL and Clo. For NFATc1 staining, NFATc1 is shown in green and DAPI‐stained nuclei in blue. For CTSK staining, CTSK is shown in red, F‐actin in green, and DAPI‐stained nuclei in blue. Scale bars, 1000 µm (C, E, upper rows), 400 µm (C, E, lower rows), 200 µm (L), and 50 µm (O). Biological replicates consisted of BMMs independently isolated from different mice (n = 3 for panels B, D, F, H–K, P and Q; n = 6 for panels M and N). Data are presented as the mean ± S.D and p values were determined using one‐way ANOVA followed by Tukey's multiple‐comparisons test(B, D, F, P, Q), unpaired two‐tailed Student's t‐test (M, N), two‐way ANOVA followed by Šídák's multiple‐comparisons test (H–K). ns, not significant; ^*^ *p* < 0.05; ^**^ *p* < 0.01.

### 3.3 Clo Inhibits RANKL‐Induced OC Differentiation In Vitro

Clo is a classical centrally acting antitussive that has been widely used in clinical practice for decades and is characterized by a favorable safety profile. This renders it a suitable candidate for drug repurposing [31]. A previous study reported that Clo reduced the abundance of several proteins associated with mitochondrial respiratory‐chain complexes I and IV, suggesting its potential to modulate mitochondrial oxidative metabolism [11]. Based on the screening results, Clo was selected for in vitro validation. CCK‐8 assays showed that Clo had minimal effects on BMMs viability at concentrations up to 10 µM, whereas clear cytotoxicity emerged from 20 µm onward (Figure 2B). To further evaluate cytotoxicity using a metabolism‐independent assay, LDH release from BMMs was measured after treatment with Clo for 48 and 96 h. Clo at concentrations up to 10 µM did not significantly increase LDH release at either time point, whereas 20 µm Clo significantly increased LDH release (Figure S3). These findings confirm that the working concentration of 10 µm did not induce detectable membrane damage or overt cytotoxicity. Within this non‐cytotoxic concentration range, Clo reduced the formation of TRAP‐positive multinucleated OCs in a dose‐dependent manner (Figure 2C,D). Moreover, time‐of‐addition experiments revealed that treatment with 10 µm Clo suppressed OC formation when administered at different stages of differentiation, with the strongest inhibitory effects observed during the early and middle phases (Figure 2E,F).

NFATc1 is the master transcription factor governing RANKL‐induced osteoclastogenesis. The transcription factor integrates upstream signaling pathways and orchestrates the expression of OC‐specific genes [32]. The transcription factor c‐Fos directly contributes to OC formation and maturation by regulating NFATc1 activation [33]. In particular, c‐Fos works in concert with integrin β3 during OC differentiation, thereby modulating both adhesion to and degradation of the extracellular matrix [34]. CTSK is a key enzyme secreted by OCs and is regulated by this molecular network, thus playing a critical role in bone matrix degradation [35]. Therefore, the effects of Clo on RANKL‐induced time‐dependent upregulation of integrin β3, CTSK, NFATc1, and c‐Fos were evaluated during differentiation (days 1–5). Western blot analyses revealed that Clo markedly suppressed integrin β3 expression at day 5, reduced NFATc1 and c‐Fos levels at days 1, 3, and 5, and decreased CTSK expression at days 3 and 5 (Figure 2G–K). Consistent with impaired OC maturation, F‐actin staining demonstrated that Clo disrupted the formation of the characteristic F‐actin belt and markedly reduced both the OC spreading area and the number of nuclei per OC (Figure 2L–N). Furthermore, immunofluorescence analysis confirmed the attenuated NFATc1 and CTSK signals in Clo‐treated cultures and revealed that Clo effectively inhibited RANKL‐induced nuclear translocation of NFATc1 (Figure 2O–Q). Collectively, these findings demonstrated that Clo robustly suppressed RANKL‐induced OC differentiation and maturation in vitro.

### 3.4 Clo Inhibits OC Differentiation by Suppressing OXPHOS

To further elucidate the mechanism underlying the Clo‐mediated inhibition of osteoclastogenesis, RNA‐seq was performed on BMM‐derived OCs stimulated with RANKL in the presence or absence of Clo (Figure 3A). Principal component analysis revealed a clear segregation between vehicle‐ and Clo‐treated samples, thereby indicating a robust and distinct transcriptional response to Clo treatment (Figure 3B). Consistently, Clo profoundly remodeled the global transcriptional landscape (Figure 3C) and substantially downregulated the expression of key osteoclastogenic genes, including *Itgb3, Ctsk, Nfatc1*, and *Fos* (Figure 3D).

**FIGURE 3:**
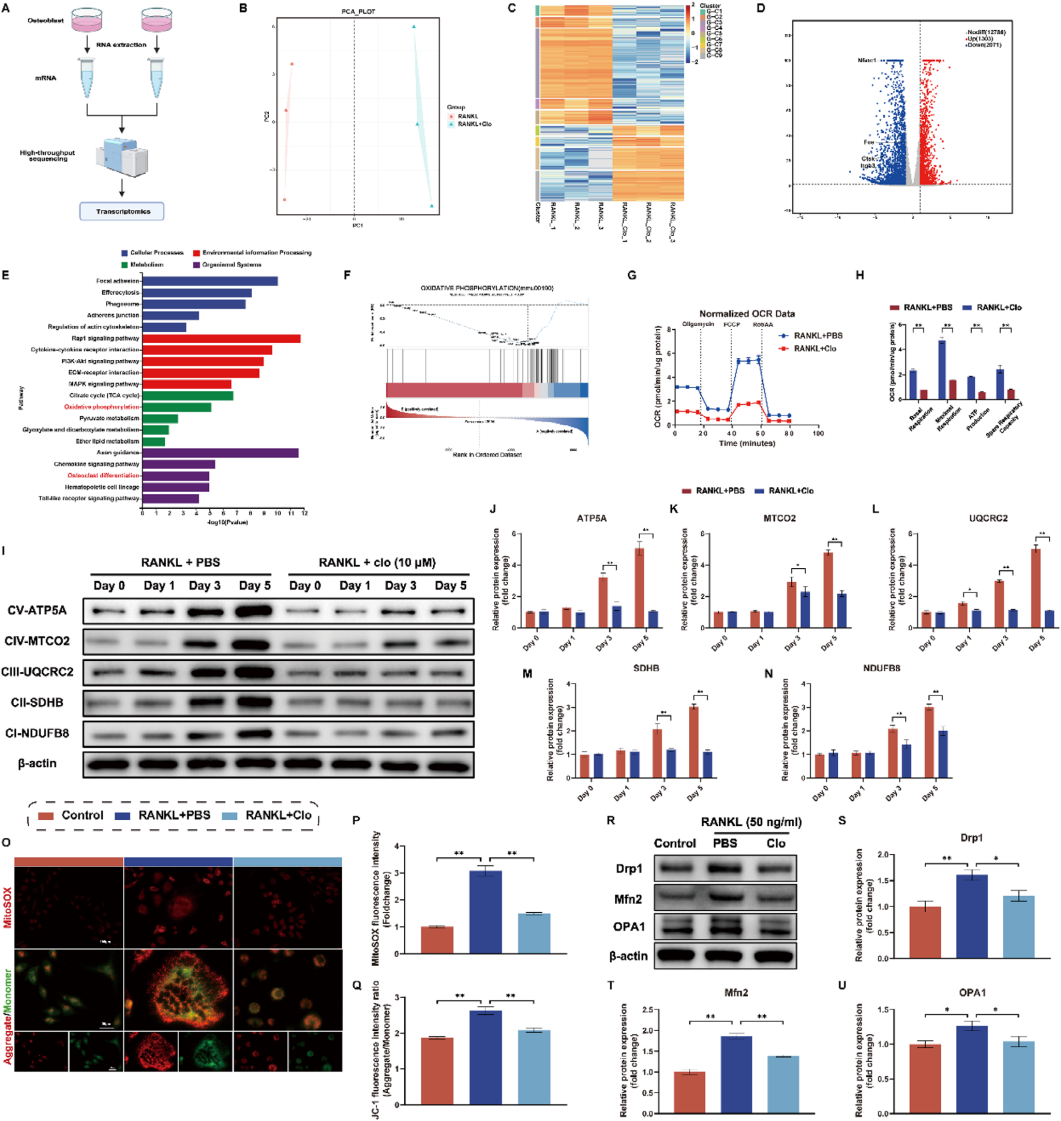
Clo inhibits osteoclast differentiation by suppressing mitochondrial oxidative phosphorylation. (A) Schematic diagram of the RNA‐seq workflow. (B) PCA of the samples. (C) Heatmap of differentially expressed genes. (D) Heatmap of differentially expressed genes showing marked downregulation of osteoclast‐specific genes *Itgb3*, *Ctsk*, *Nfatc1*, and *Fos*. (E) KEGG pathway enrichment analysis. (F) GSEA results showing that Clo suppresses OXPHOS in OCs. (G, H) Seahorse XF96 mitochondrial stress test indicates that Clo suppresses OXPHOS in OCs. (I–N) Western blot analysis and quantification of mitochondrial OXPHOS complex proteins in cells treated with RANKL (50 ng/mL) and Clo (10 µm) for 0, 1, 3, and 5 days. (O–Q) Detection of mitochondrial reactive oxygen species and membrane potential in osteoclasts using MitoSOX and JC‐1 probes, with or without RANKL and Clo treatment. MitoSOX fluorescence is shown in red; JC‐1 aggregates and monomers are shown in red and green, respectively. Scale bars, 100 µm (MitoSOX) and 20 µm (JC‐1) (R–U) Western blot analysis of mitochondrial dynamics–related proteins in cells treated with or without RANKL and Clo. All experiments used three biological replicates per group, each derived from BMMs independently isolated from a different mouse. Data are presented as the mean ± S.D and p values were determined using unpaired two‐tailed Student's t‐test (H), two‐way ANOVA followed by Šídák's multiple‐comparisons test (J–N), and one‐way ANOVA followed by Tukey's multiple‐comparisons test (P, Q, S, T, U). ns, not significant; ^*^ *p* < 0.05; ^**^ *p* < 0.01.

The current study findings and those of previous studies highlight the essential role of OXPHOS as a primary energy source during OC differentiation. A previous study reported that Clo reduced the abundance of several proteins associated with mitochondrial respiratory‐chain complexes I and IV, including NDUFA1, NDUFS5, and COX6B1, suggesting that Clo may affect mitochondrial oxidative metabolism [11]. In the present study, KEGG pathway enrichment analysis of the RNA‐seq data identified OXPHOS as one of the most substantially altered pathways following Clo treatment (Figure 3E). Moreover, GSEA revealed a pronounced negative enrichment of the OXPHOS pathway in Clo‐treated OCs, whereas glycolysis‐related gene sets remained largely unchanged (Figure 3F, Figure S4A). Consistently, Clo treatment did not markedly alter the expression of HK2, PKM2, or LDHA (Figure S4B–E). This suggests that Clo preferentially disrupted mitochondrial respiration rather than glycolytic metabolism.

To validate these transcriptomic findings functionally, mitochondrial bioenergetics were assessed using the Seahorse XF96 mitochondrial stress test. Clo markedly reduced basal respiration, ATP‐linked respiration, maximal respiratory capacity, and spare respiratory capacity in RANKL‐stimulated OCs (Figure 3G,H), indicating impaired mitochondrial OXPHOS. Similar effects were observed in BMMs cultured without RANKL, in which Clo markedly reduced basal and maximal OCR (Figure S5). To further assess respiratory‐chain function, complex I enzymatic activity was measured. RANKL significantly increased complex I activity, whereas Clo significantly attenuated this increase. Clo also significantly reduced complex I activity in the absence of RANKL (Figure S6). These findings suggest that the mitochondrial effects of Clo are not solely attributable to impaired RANKL‐induced OC differentiation and may contribute to its anti‐osteoclastogenic effects. Consistent with these functional defects, time‐course western blot analyses revealed that RANKL stimulation progressively increased the abundance of representative subunits of OXPHOS complexes *I–V* during OC differentiation, whereas Clo treatment substantially attenuated these increases on days 3 and 5 (Figure 3I,N). Collectively, these results show that Clo treatment reduces mitochondrial respiratory capacity and complex I enzymatic activity, accompanied by a coordinated decrease in representative OXPHOS subunit abundance. Whether the reduction in complex I activity reflects a primary effect on the respiratory chain or secondary mitochondrial changes remains to be clarified.

Given that mitochondrial homeostasis is tightly coupled to OC differentiation [36], mitochondrial quality and function were evaluated. RANKL stimulation markedly increased mitochondrial ROS production and sustained a high mitochondrial membrane potential in differentiated OCs. In contrast, Clo treatment markedly reduced MitoSOX fluorescence and decreased the JC‐1 red/green ratio. This indicated diminished mitochondrial ROS levels and the dissipation of mitochondrial membrane potential (Figure 3O–Q). Furthermore, Clo altered the expression patterns of proteins involved in mitochondrial dynamics, thus reversing or attenuating RANKL‐induced changes in key fusion and fission regulators (Figure 3R–U). These findings suggest that Clo modulates mitochondrial dynamics and disrupts mitochondrial homeostasis. Collectively, these data support the conclusion that the inhibitory effect of Clo on OC differentiation is accompanied by impaired mitochondrial OXPHOS, altered membrane potential, and remodeling of mitochondrial homeostasis.

### 3.5 Clo Treatment Attenuates OVX‐Induced Bone Loss and Inhibits OC Activity in Mice

To investigate the protective effects of Clo against OVX‐induced bone loss, OVX mice were used as an experimental model. Clo treatment was initiated one week after OVX and administered intravenously every two days at doses of 5 and 10 mg/kg until the sixth week. The representative micro‐CT reconstruction images (Figure S7A) showed that both Clo (5 mg/kg) and Clo (10 mg/kg) treatments effectively mitigated OVX‐induced bone loss compared with that in the OVX group. Analysis of bone microarchitecture (Figure S7B–E) revealed improvements in BV/TV, Tb.N, and Tb.Sp at both doses, with no notable differences between them. H&E staining (Figure S7F) confirmed well‐preserved bone structure in the Clo‐treated groups. TRAP staining and quantitative analysis (Figure S7G–I) showed reduced N.Oc/BS and Oc.S/BS in both Clo groups, which indicated the inhibition of OC activity. As the two doses exhibited similar protective effects, a lower dose (5 mg/kg) was selected for subsequent in vivo studies to minimize drug exposure while maintaining efficacy.

### 3.6 Preparation and Characterization of ROS‐Responsive Clo‐Loaded Asp8‐PMK‐MCs

Although the results demonstrate that Clo functions as an OXPHOS inhibitor that suppresses OC differentiation, concerns regarding systemic exposure and potential off‐target effects remain. As a classical antitussive approved for pediatric use, Clo exhibits an overall favorable safety profile at therapeutic doses and preclinical toxicological studies have not reported overt toxicity [31]. Nevertheless, excessive Clo administration in animal models has been associated with QT interval prolongation, which raises concerns about the increased risk of arrhythmia [37]. In addition, rare clinical case reports have described acute dystonic reactions following Clo syrup ingestion, which are potentially linked to pre‐existing neurological susceptibility [38]. Therefore, to enhance skeletal accumulation, while minimizing extraskeletal exposure, a bone‐targeted nanocarrier for Clo delivery was developed.

Asp8 is a short oligopeptide composed of eight aspartic acid residues carrying a strong net negative charge, thereby enabling stable electrostatic binding to hydroxyapatite. Owing to this property, Asp8 has been widely employed as a bone‐targeting ligand to enhance drug accumulation in bone tissue [39]. OC differentiation is accompanied by substantial ROS production, which is recognized as a critical regulator of osteoclastogenesis and bone resorption [40]. Based on these considerations, an ROS‐responsive polymethionine/lysine block copolymer was selected as the carrier backbone to enable ROS‐triggered micellar disassembly and controlled drug release. Several essential amino acids, including lysine, threonine, methionine, tryptophan, and isoleucine, have been implicated in the regulation of bone homeostasis [41]. Methionine is closely linked to endogenous antioxidant capacity, inhibits OC differentiation, and participates in bone remodeling regulation [42, 43]. In contrast, lysine has been associated with enhanced OB differentiation [44]. Collectively, these considerations provided a strong rationale for designing bone‐targeted and ROS‐responsive nanoplatforms for Clo delivery.

Thereafter, an ROS‐responsive Clo delivery system was constructed based on mPEG‐PMK micelles. Specifically, mPEG‐PMK, MAL‐mPEG‐PMK, and Asp8‐mPEG‐PMK were synthesized stepwise, as described in the Methods section, and their chemical structures were confirmed via ^1^H NMR spectroscopy. The proton resonance signals of mPEG‐PMK, MAL‐mPEG‐PMK, and Asp8‐mPEG‐PMK exhibited common characteristics at 4.01–4.47 ppm (a, −CO−CH‐(CH−)−NH−), 7.97 ppm (b, −CH(CH2−)−NH−), 2.01 ppm (c, −CH2−CH2−S−CH3), 2.50 ppm (d, −CH2−CH2−S−CH3), 1.45 ppm (e, −CH2−CH2−CH2−), 3.13 ppm (f, −CH2−CH2−NH−), and 9.38 ppm (g, −NH−CO−CF3) (Figure 4A, B, Figure S8). The characteristic signal of the 2, 5‐dioxo‐2, 5‐dihydropyrrole group at 6.99 ppm (i, −CO−CH = CH−CO−) confirmed the successful synthesis of MAL‐mPEG‐PMK (Figure S8E). The disappearance of the 6.99 ppm peak indicated that Asp8 was successfully linked to MAL, thus confirming the successful synthesis of Asp8‐mPEG‐PMK (Figure S8F). Based on the integral ratio of the polypeptide α‐CH signals at 4.01–4.47 ppm to the PEG backbone signals at 3.40–3.75 ppm, the total degrees of polymerization of the PMK segments were estimated to be approximately 53.0 for mPEG‐PMK and 51.8 for MAL‐mPEG‐PMK. Using the designed Met: Lys(TFA) molar ratio of 18:30, the corresponding ^1^H NMR‐estimated number‐average molecular weights (M_n, NMR_) were both approximately 15.0 kDa (Table S6). GPC‐derived molecular‐weight distributions and dispersities were not obtained because of the insufficient solubility of these polymers in the available GPC mobile phase. Upon incubation with 0.1 or 10 mm H_2_O_2_ for 24 h, the hydrophobic thioether groups in Asp8‐mPEG‐PMK were oxidized to hydrophilic sulfoxide or a sulfone group, as evidenced by a characteristic downfield shift of the corresponding proton signals (from 2.15 to 2.77 ppm) (Figure 4C). This confirmed the ROS‐responsive nature of the polymer.

**FIGURE 4:**
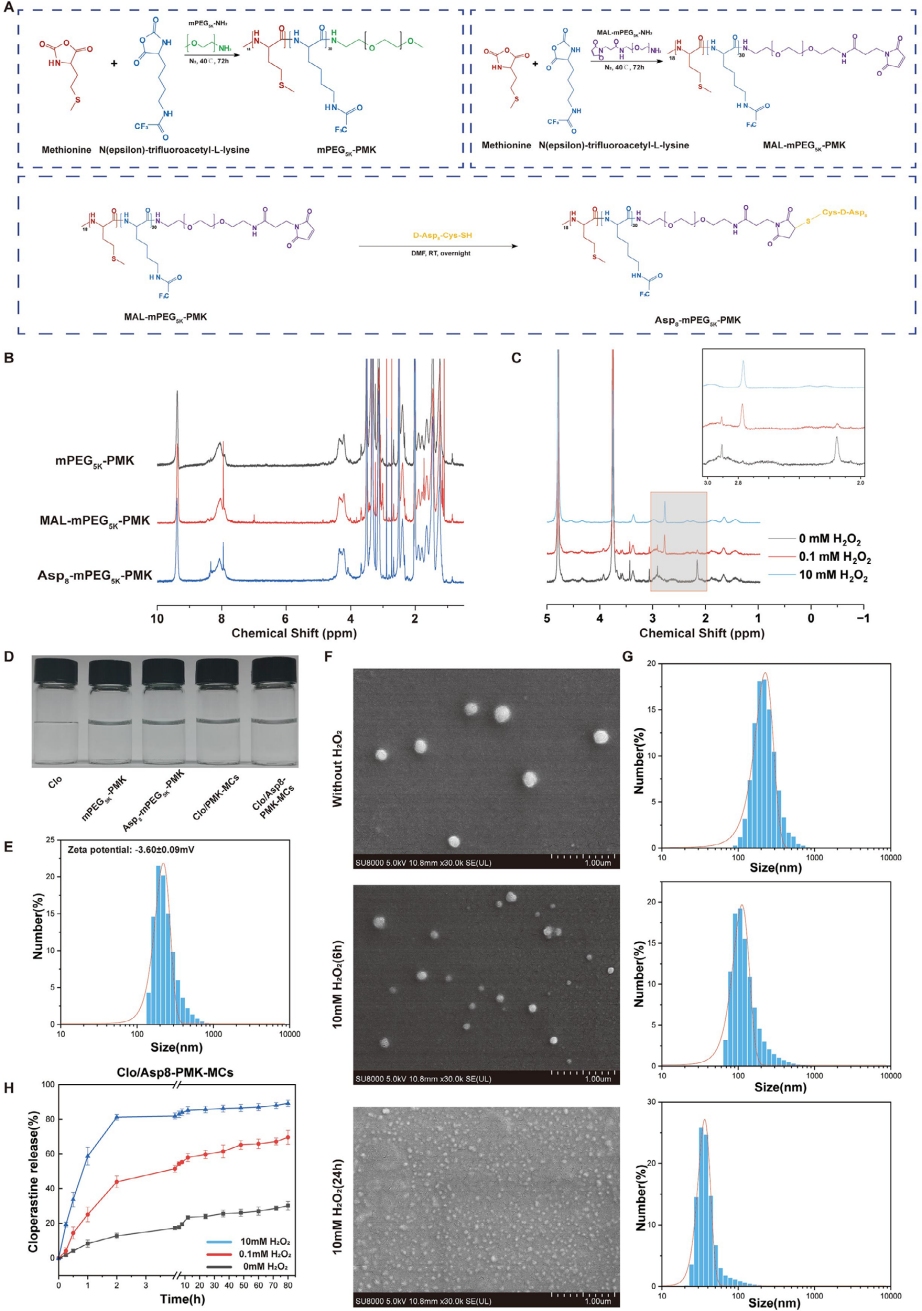
Preparation and characterization of mPEG‐PMK, MAL‐mPEG‐PMK, Asp8‐mPEG‐PMK, and Clo‐loaded PMK‐MCs. (A) Synthesis procedure of mPEG‐PMK, MAL‐mPEG‐PMK, and Asp8‐mPEG‐PMK. (B) ^1^H NMR spectra of mPEG‐PMK, MAL‐mPEG‐PMK, and Asp8‐mPEG‐PMK. (C) The thioether group of Asp8‐mPEG‐PMK was oxidized to sulfoxide after 24 h incubation in 0.1 or 10 mm H_2_O_2_, as confirmed by ^1^H NMR analysis. (D) Appearance of Clo, mPEG‐PMK, Asp8‐mPEG‐PMK, Clo/PMK‐MCs, and Clo/Asp8‐PMK‐MCs in distilled water. (E) Size and zeta potential of Clo/Asp8‐PMK‐MCs in distilled water. (F, G) SEM images and size changes of Clo/Asp8‐PMK‐MCs after incubation for 6 and 24 h with or without 10 mm H_2_O_2_. Scale bars, 1 µm. (H) In vitro release of Clo from Clo/Asp8‐PMK‐MC solution in the presence or absence of 0.1 and 10 mm H_2_O_2_. For panels E, G and H, n = 3 independently prepared formulation batches.

In distilled water, Clo/Asp8‐PMK‐MCs formed a clear and stable nanoscale dispersion (Figure 4D) with an average hydrodynamic diameter of approximately 220 nm and a zeta potential of −3.60 ± 0.09 mV (Figure 4E). DLS and SEM analyses showed consistent particle sizes, and the SEM images revealed uniformly spherical nanoparticles under non‐oxidative conditions (Figure 4F,G). In contrast, after incubation with 10 mm H_2_O_2_ for 6 or 24 h, the micelles exhibited pronounced structural disruption and fragmented into smaller particles, thereby indicating ROS‐triggered micellar disassembly (Figure 4F,G). Drug‐loading analysis via fluorescence spectroscopy demonstrated an encapsulation efficiency of 84.17% and a drug‐loading content of 7.11%. Furthermore, in vitro release studies showed sustained Clo release under non‐oxidative conditions, whereas exposure to 0.1 or 10 mm H_2_O_2_ markedly accelerated Clo release in an oxidant concentration‐dependent manner (Figure 4H). This confirmed efficient ROS‐responsive drug release from the micellar system. The size stability of Clo/Asp8‐PMK‐MCs was evaluated in ddH_2_O, PBS, and serum conditions and demonstrated consistent particle sizes across these environments (Figure S9).

To verify whether the osteoporotic bone microenvironment provided sufficient oxidative stress to activate the ROS‐responsive nanoplatform, dihydroethidium (DHE) staining was performed on femoral bone sections from Sham and OVX mice. As shown in Figure S10, the DHE fluorescence intensity was markedly increased in the trabecular bone of OVX mice, thus indicating elevated ROS levels under osteoporotic conditions. This observation is consistent with those of previous studies showing that osteoporotic bone exhibits an oxidative microenvironment. This provides a pathological basis for ROS‐responsive biomaterials and nanocarriers to achieve disease‐specific activation [45]. Although the precise local ROS concentration could not be directly quantified in vivo, these results support the existence of an oxidative microenvironment capable of facilitating ROS‐responsive micelle activation.

### 3.7 Clo/Asp8‐PMK‐MCs Show Acceptable Cytocompatibility Toward OC Precursors and OBs

Bone remodeling is governed by the coordinated activities of OCs and OBs [46]. OCs originate from the precursor cells of the Mono/macrophage lineage and undergo differentiation in response to RANKL and M‐CSF stimulation [47]. First, the cytotoxicity of Clo/Asp8‐PMK‐MCs was evaluated in BMMs. CCK‐8 assays demonstrated that Clo/Asp8‐PMK‐MCs did not substantially reduce cell viability at 10 µm, which was the concentration used in subsequent experiments (Figure S11A). Consistently, Calcein AM/PI staining revealed no evident increase in dead cells following treatment with Clo, Asp8‐PMK‐MCs, or Clo/Asp8‐PMK‐MCs. This indicated favorable biocompatibility of the micellar formulation toward OC precursors (Figure S11B,C).

Next, the cytocompatibility of the OBs was assessed. Neither free Clo nor Clo/Asp8‐PMK‐MCs caused a detectable reduction in OB viability, even at the highest concentrations tested (Figure S20A,B). Similarly, Calcein AM/PI staining showed no apparent increase in OB death after exposure to Clo, Asp8‐PMK‐MCs, or Clo/Asp8‐PMK‐MCs (Figure S20C). Collectively, these results indicated that both free Clo and Clo/Asp8‐PMK‐MCs exhibited minimal cytotoxicity toward OC precursors and OBs within the experimental dose range.

### 3.8 In Vivo Fluorescence Tracking and In Vitro Cellular Uptake of Asp8‐PMK‐MCs

In vivo fluorescence imaging was performed to compare the bone‐targeting efficiency of DID‐labeled Asp8‐PMK‐MCs (DID/Asp8‐PMK‐MCs) with that of non‐targeted DID/PMK‐MCs. DID/Asp8‐PMK‐MCs showed stronger fluorescence signals in skeletal regions than DID/PMK‐MCs, consistent with enhanced skeletal localization of the labeled formulation following Asp8 modification (Figure S12A). These findings provide preliminary support for the bone‐targeting potential of the Asp8‐modified carrier system. However, because these measurements track the fluorescent label rather than Clo itself, they provide indirect evidence for bone‐directed drug delivery. Direct quantitative measurements of Clo in bone, plasma, and major organs over time following administration of free Clo and Clo/Asp8‐PMK‐MCs are needed to determine whether, and to what extent, the observed carrier localization translates into increased bone exposure to Clo.

To further assess cellular uptake, DID/Asp8‐PMK‐MCs were incubated with BMMs and OBs for 1, 3, or 6 h. In both cell types, the intracellular fluorescence intensity increased in a time‐dependent manner, with the highest signal observed at 6 h. This indicated sustained internalization of the nanocarriers (Figure S12B,C). Collectively, these results support enhanced skeletal accumulation following Asp8 modification and uptake of the labeled micelles by BMMs and OBs in vitro, but do not demonstrate selective localization to OC‐rich regions in vivo.

Asp8 has been primarily utilized as a bone‐targeting ligand in nanomedicine applications, and its potential intrinsic effects on cellular differentiation are considered limited [48]. To further determine whether Asp8 modification influenced OC or OB differentiation, the biological effects of PMK‐MCs and Asp8‐PMK‐MCs were compared. Both PMK‐MCs and Asp8‐PMK‐MCs exhibited modest inhibitory effects on OC differentiation, whereas no pronounced difference in TRAP‐positive OC formation was observed between the two groups (Figure S13A,B). Similarly, ALP staining (Figure S13C,D), and ARS staining (Figure S13E,F) demonstrated that both nanocarriers exhibited pro‐osteogenic effects and promoted matrix mineralization, with no considerable differences between PMK‐MCs and Asp8‐PMK‐MCs. These results indicated that Asp8 modification did not significantly affect the intrinsic biological activities of the PMK‐based nanocarrier in regulating OC or OB differentiation. Therefore, the modest anti‐osteoclastogenic and pro‐osteogenic effects observed for both PMK‐MCs and Asp8‐PMK‐MCs were more likely attributable to the methionine‐ and lysine‐based polymeric components of the PMK carrier rather than the Asp8 peptide itself. Given the similar biological activities between PMK‐MCs and Asp8‐PMK‐MCs, subsequent studies focused on Asp8‐PMK‐MCs to evaluate the therapeutic effects of the Clo‐loaded formulation.

### 3.9 Clo/Asp8‐PMK‐MCs Inhibit OC Differentiation and Function

The study investigated the anti‐osteoclastic effects of Clo/Asp8‐PMK‐MCs and the contribution of the nanocarrier system. Following RANKL stimulation, treatment with Clo/Asp8‐PMK‐MCs markedly reduced the number and size of TRAP‐positive multinucleated OCs, thus indicating a pronounced inhibitory effect on OC differentiation (Figure 5A,B).

**FIGURE 5:**
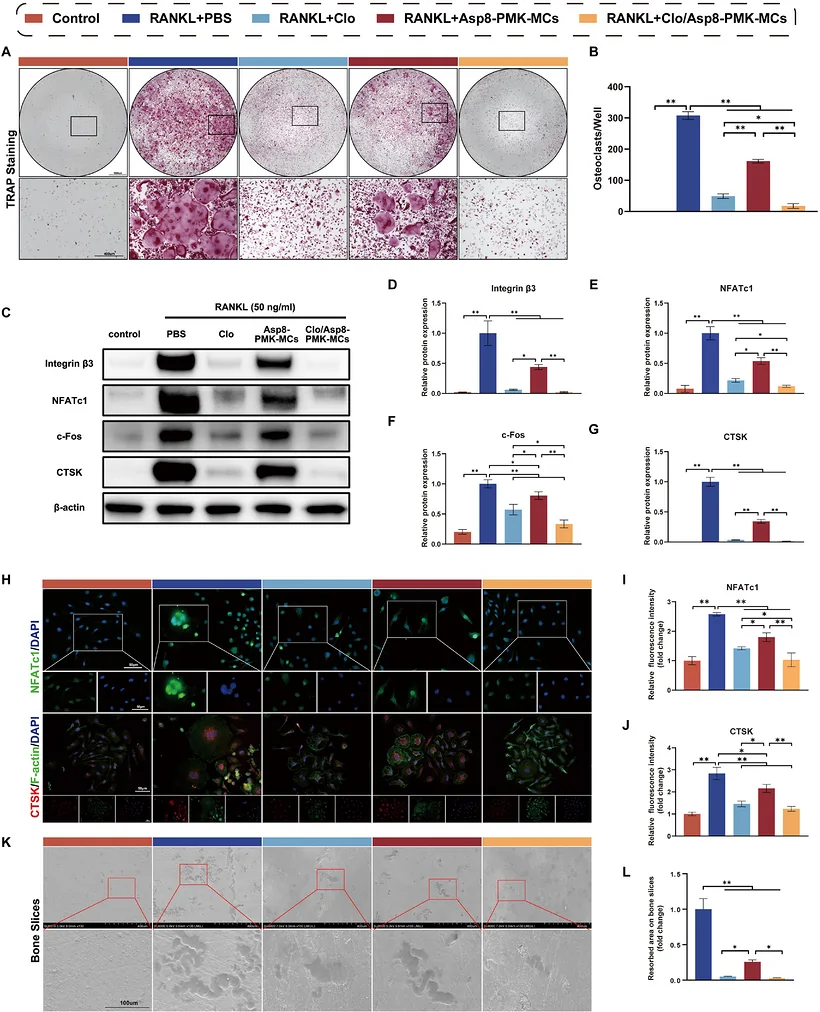
Clo/Asp8‐PMK‐MCs suppressOC differentiation in vitro. (A, B) TRAP staining images and quantitative analysis of OC differentiation after 5 days of treatment with Clo, Asp8‐PMK‐MCs, or Clo/Asp8‐PMK‐MCs under RANKL stimulation. (C–G) Representative Western blot images and quantitative analysis of integrin β3, CTSK, NFATc1, and c‐Fos under different conditions. (H–J) Immunofluorescence images and quantitative analysis of NFATc1 and CTSK in osteoclasts under different conditions. For NFATc1 staining, NFATc1 is shown in green and DAPI‐stained nuclei in blue. For CTSK staining, CTSK is shown in red, F‐actin in green, and DAPI‐stained nuclei in blue. (K, L) Effects of Clo, Asp8‐PMK‐MCs, or Clo/Asp8‐PMK‐MCs on bone resorption by OCs. Scale bars, 1000 µm (A, upper row), 400 µm (A, lower row; K, upper row), 50 µm (H), and 100 µm (K, lower row). All experiments used three biological replicates per group, each derived from BMMs independently isolated from a different mouse. Data are presented as the mean ± S.D and p values were determined using one‐way ANOVA followed by Tukey's multiple‐comparisons test. ns, not significant; ^*^ *p* < 0.05; ^**^ *p* < 0.01.

At the molecular level, Clo/Asp8‐PMK‐MCs inhibited the expression of osteoclastogenic markers. Among the proteins examined, NFATc1 and c‐Fos were markedly downregulated, whereas integrin β3 and CTSK showed a decreasing trend that did not reach statistical significance (Figure 5C–G). The RT‐qPCR analysis consistently showed similar expression patterns at the mRNA level (Figure S14). Immunofluorescence staining further confirmed the reduced NFATc1 and CTSK fluorescence signals in the Clo/Asp8‐PMK‐MC–treated group; however, the magnitude of the change was relatively modest (Figure 5H–J). Functionally, Clo/Asp8‐PMK‐MCs markedly attenuated OC resorptive activity as evidenced by reduced resorption pit formation (Figure 5K,L). In parallel, Clo/Asp8‐PMK‐MCs disrupted F‐actin belt formation, decreased OC spreading and multinucleation, and reduced intracellular ROS levels (Figure S15A–E). This indicated impaired cytoskeletal maturation and bone resorption. Collectively, these results demonstrate that Clo/Asp8‐PMK‐MCs effectively suppress OC differentiation and resorptive function. Together with the effects observed for blank Asp8‐PMK‐MCs, these findings suggest that the anti‐osteoclastogenic activity of the nanoplatform may involve both Clo‐mediated inhibition and modest carrier‐associated regulation.

### 3.10 Clo/Asp8‐PMK‐MCs Suppress Mitochondrial OXPHOS in OCs

Under RANKL stimulation, Seahorse XF96 analysis revealed that all treatment groups exhibited reduced mitochondrial respiration compared with that of the PBS control. Blank Asp8‐PMK‐MCs induced a modest decrease in maximal respiration and spare respiratory capacity (Figure 6A,B). In contrast, both Clo and Clo/Asp8‐PMK‐MCs caused more pronounced suppression of OXPHOS parameters, including basal respiration, maximal respiration, ATP‐linked respiration, and spare respiratory capacity. Consistent with the functional respiratory data, western blot analysis showed that RANKL stimulation increased the expression of mitochondrial OXPHOS complex proteins, whereas this upregulation was markedly attenuated by Clo and Clo/Asp8‐PMK‐MCs after five days of treatment (Figure 6C–H). In contrast, Asp8‐PMK‐MCs alone produced only a mild reduction in the OXPHOS complex protein levels, which was substantially weaker than that observed in the Clo‐containing groups.

**FIGURE 6:**
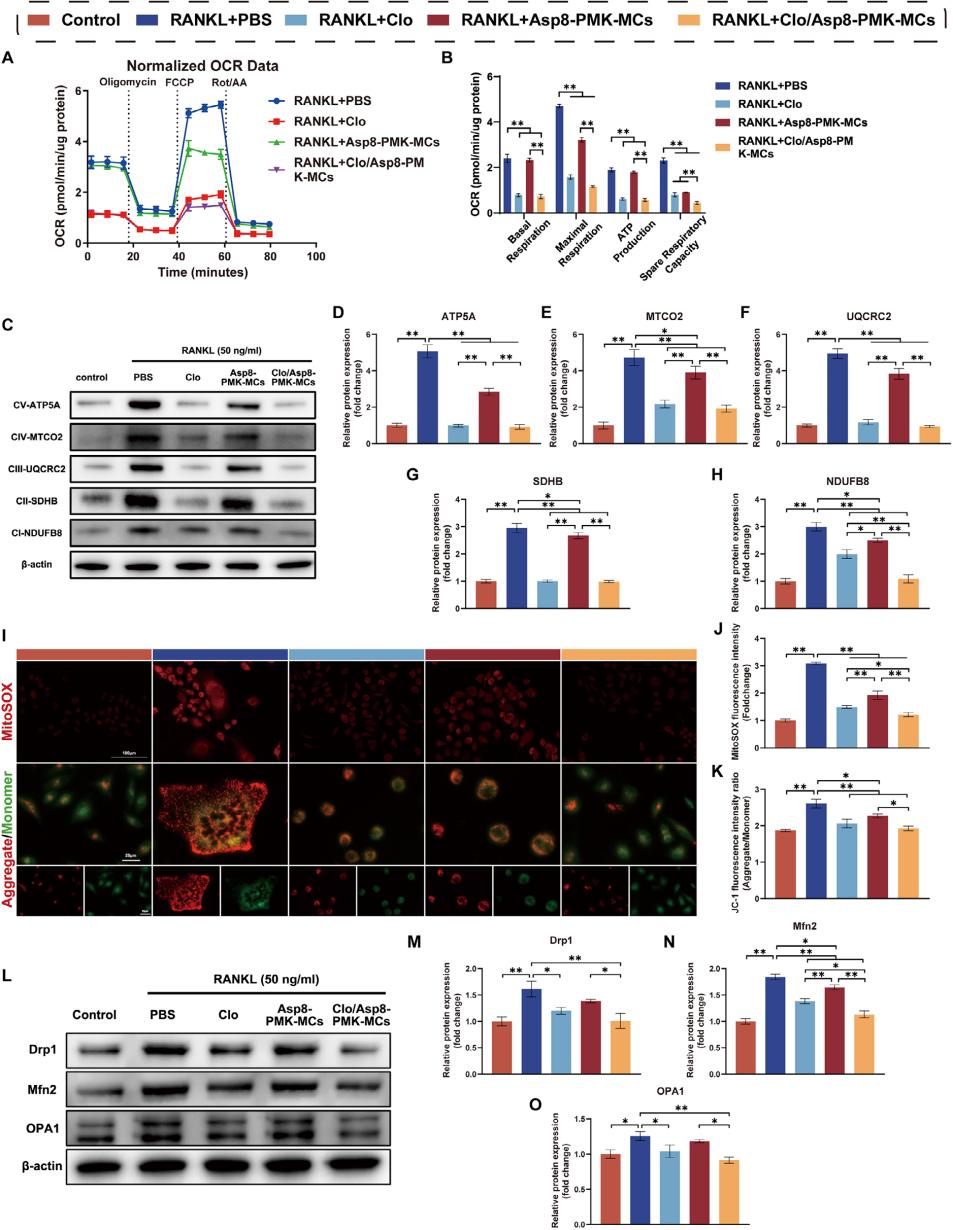
Clo/Asp8‐PMK‐MCs inhibit OC differentiation by suppressing mitochondrial oxidative phosphorylation. (A, B) Seahorse XF96 mitochondrial stress test shows that Clo/Asp8‐PMK‐MCs further suppress OXPHOS in OCs. (C–H) Under RANKL stimulation, western blot analysis of mitochondrial OXPHOS complex protein levels after 5 days of co‐incubation with Clo, Asp8‐PMK‐MCs, or Clo/Asp8‐PMK‐MCs. (I–K) MitoSOX and JC‐1 probes were used to detect mitochondrial ROS and membrane potential in OCs under different conditions. MitoSOX fluorescence is shown in red; JC‐1 aggregates and monomers are shown in red and green, respectively. Scale bars, 100 µm (MitoSOX) and 20 µm (JC‐1). (L–O) Western blot analysis of mitochondrial dynamics–related protein expression in OCs under different conditions. All experiments used three biological replicates, each derived from BMMs independently isolated from a different mouse. Data are presented as the mean ± S.D and p values were determined using two‐way ANOVA followed by Šídák's multiple‐comparisons test (B), one‐way ANOVA followed by Tukey's multiple‐comparisons test (D–H, J, K, M–O). ns, not significant; ^*^ *p* < 0.05; ^**^ *p* < 0.01.

MitoSOX and JC‐1 staining further demonstrated that both Clo and Clo/Asp8‐PMK‐MCs substantially reduced mitochondrial ROS production and dissipated the mitochondrial membrane potential compared with that of RANKL stimulation alone. In contrast, Asp8‐PMK‐MCs exerted only relatively modest effects (Figure 6I–K). Correspondingly, Clo and Clo/Asp8‐PMK‐MCs altered the expression of proteins involved in mitochondrial dynamics by partially reversing RANKL‐induced changes, whereas blank micelles showed only a minimal impact (Figure 6L–O). Collectively, these results indicated that Clo retained its inhibitory effects on mitochondrial OXPHOS and homeostasis after encapsulation in Asp8‐PMK micelles. Notably, these findings provide initial evidence that a polymethionine/lysine‐based polymer can influence mitochondrial homeostasis. This highlights the need for further investigation into the mechanisms by which essential amino acid–derived biomaterials modulate OC biology.

### 3.11 PGC1β is a Potential Molecular Target of Clo in OCs

Members of the PGC1 family are key regulators of mitochondrial biogenesis and oxidative metabolism, among which PGC1β appears to play a predominant role in OCs [49]. The preferential involvement of PGC1β in OCs may be attributed to the distinct metabolic requirements of bone cell lineages. In particular, OCs rely on PGC1β‐associated OXPHOS to meet the high energetic demand of differentiation and bone‐resorptive activity, whereas OBs exhibit a more balanced metabolic profile and appear less dependent on PGC1β‐mediated mitochondrial regulation [50, 51]. Consistently, Human Protein Atlas data suggest higher PGC1β expression in Mono/macrophage lineages than in OB‐related cells (Figure S16). RNA‐seq analysis showed that *Ppargc1a* expression was barely detectable, whereas *Ppargc1b* (PGC1β) was robustly expressed and markedly downregulated upon Clo treatment (Figure S17A). This suggests the involvement of PGC1β in Clo‐mediated regulation.

To explore whether Clo potentially interacts with PGC1β, molecular docking was performed using the available crystal structure of the PPAR–PGC1β coactivator complex (PDB ID: 3SP6). This complex represents the nuclear receptor–coactivator interaction interface mediated by the PGC1β LXXLL motif. The docking analysis predicted a favorable binding mode with a predicted binding energy of −8.0 kcal/mol. Detailed interaction analysis indicated that Clo is stabilized within a protein–protein interaction interface through hydrogen bonding and hydrophobic interactions. Specifically, Clo formed a hydrogen bond with ALA333, whereas hydrophobic interactions with ILE272, CYS278, ILE339, and LEU344 stabilized the predicted binding conformation (Figure S17B–D). Functionally, western blot analysis demonstrated that Clo reduced PGC1β protein levels in RANKL‐stimulated OCs (Figure S17E,F). Moreover, CETSA results revealed changes in the thermal stability profile of PGC1β in the presence of Clo. This suggests potential alterations in protein stability or protein complex‐associated effects rather than direct ligand–protein binding (Figure S17G,H). Similarly, Clo/Asp8‐PMK‐MCs substantially decreased PGC1β expression, whereas Asp8‐PMK‐MCs alone induced a modest but statistically significant reduction (Figure S17I,J). This indicated that nanocarrier encapsulation preserves the ability of Clo to modulate PGC1β.

To further investigate the functional role of PGC1β in Clo‐mediated inhibition of osteoclastogenesis, a lentiviral PGC1β overexpression system was established. RT‐qPCR and western blot analyses confirmed successful overexpression of PGC1β at both the mRNA and protein levels (Figure S18A–E). Functionally, TRAP staining demonstrated that PGC1β overexpression partially rescued the inhibitory effect of Clo on OC differentiation, as evidenced by an increased number of TRAP‐positive multinucleated OCs compared with that in the Clo‐treated group (Figure S18F,G). Conversely, siRNA‐mediated PGC1β knockdown suppressed RANKL‐induced OC formation and mitochondrial respiration, phenocopying the effects of Clo. In PGC1β‐depleted cells, Clo did not further significantly reduce OC formation, while mitochondrial respiration remained similarly low (Figure S19). Together, these complementary gain‐ and loss‐of‐function findings support PGC1β as a functional mediator of Clo‐induced inhibition of OXPHOS and osteoclastogenesis.

Overall, these findings identify PGC1β as a key functional mediator of the anti‐osteoclastogenic effects of Clo and support its potential role as a molecular target. The docking analysis was performed using the available structure of the PPAR–PGC1β coactivator complex, and the current evidence is based on molecular docking, CETSA, protein expression, and complementary gain‐ and loss‐of‐function experiments. Therefore, further biophysical studies are required to confirm direct physical binding between Clo and PGC1β.

### 3.12 Clo/Asp8‐PMK‐MCs Enhance OB Differentiation and Mineralization

The effects of Clo, Asp8‐PMK‐MCs, and Clo/Asp8‐PMK‐MCs on OB differentiation and function were examined. After seven days of osteogenic induction, ALP staining and quantitative analysis revealed that Asp8‐PMK‐MCs markedly enhanced ALP activity in OBs, whereas free Clo did not markedly increase ALP levels compared with that in the positive control group (Figure S21A,B). Treatment with Clo/Asp8‐PMK‐MCs exhibited a pro‐osteogenic effect comparable with that of Asp8‐PMK‐MCs alone. These results suggest that the enhanced osteogenic activity of Clo/Asp8‐PMK‐MCs is mainly derived from the intrinsic biological activity of the Asp8‐PMK carrier, while Clo loading does not interfere with this carrier‐mediated osteogenic effect.

Consistent with the ALP results, ARS staining after 21 days of osteogenic induction revealed substantially enhanced matrix mineralization in the Asp8‐PMK‐MC and Clo/Asp8‐PMK‐MC groups, whereas Clo alone did not promote mineral nodule formation (Figure S21C,D). Furthermore, western blot analysis revealed that the protein expression of COL1A1, RUNX2, and OCN was markedly upregulated in the Asp8‐PMK‐MC and Clo/Asp8‐PMK‐MC groups, but not in the Clo‐treated group (Figure S21E–H). Collectively, these findings demonstrate that the PMK‐based carrier provides intrinsic pro‐osteogenic activity, whereas Clo primarily contributes to anti‐osteoclastogenic effects. Therefore, the dual bone‐protective activity of Clo/Asp8‐PMK‐MCs likely results from the complementary functions of the drug and carrier components.

### 3.13 Clo/Asp8‐PMK‐MCs Improve Bone Remodeling in OVX Mice

Considering that Clo alleviates OVX‐induced bone loss in vivo, and that the Asp8‐PMK carrier itself exhibits potential bone‐regulatory activity, the study investigated whether bone‐targeted Clo/Asp8‐PMK‐MCs could further improve skeletal outcomes. Micro‐CT analysis of the distal femurs revealed pronounced trabecular bone loss in OVX mice compared with that in Sham controls, whereas treatment with Clo, Asp8‐PMK‐MCs, or Clo/Asp8‐PMK‐MCs attenuated trabecular microarchitectural deterioration (Figure 7B). Quantitative analysis showed that OVX markedly reduced BV/TV and Tb.N and increased Tb.Sp. Treatment with Asp8‐PMK‐MCs alone resulted in a measurable improvement in these parameters, suggesting that the carrier system itself contributes to the regulation of bone remodeling (Figure 7C–F). Notably, Clo/Asp8‐PMK‐MCs showed the greatest preservation of trabecular bone mass and architecture, with higher BV/TV, Tb.N, and Tb.Th and lower Tb.Sp than those observed in the free Clo group. These findings indicate that Clo/Asp8‐PMK‐MCs attenuated OVX‐induced trabecular bone loss and improved trabecular microarchitecture more than free Clo.

**FIGURE 7:**
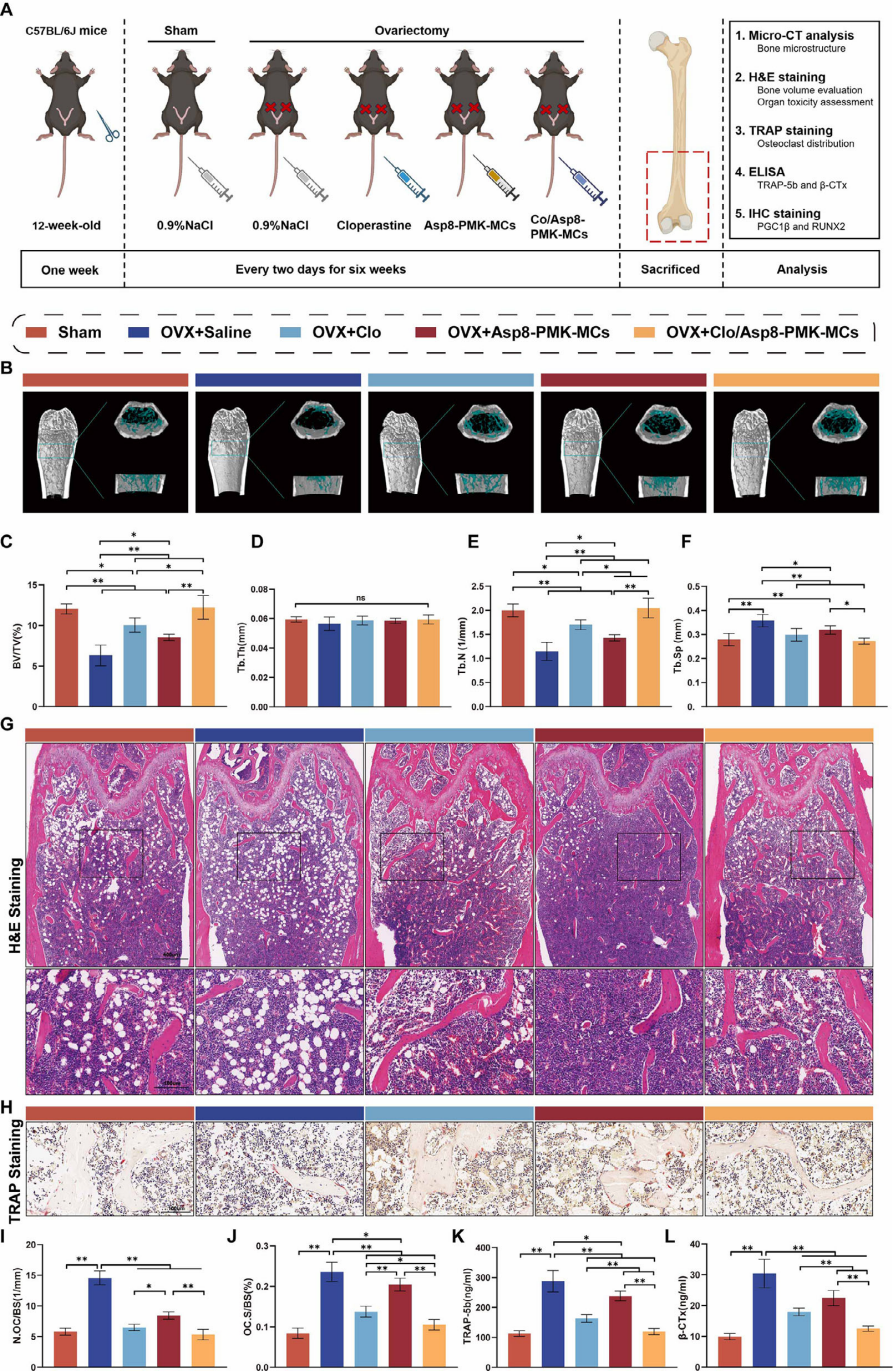
Clo/Asp8‐PMK‐MCs improve bone density in ovariectomized mice. (A) Schematic diagram of the ovariectomy model establishment and subsequent evaluations. Created using BioRender. (B) Representative two‐dimensional and three‐dimensional CT reconstruction images of the distal femur in the Sham, OVX, Clo, Asp8‐PMK‐MCs, and Clo/Asp8‐PMK‐MCs groups. (C–F) Analysis of bone microstructure parameters in each group, including trabecular BV/TV, Tb.N, Tb.Sp, and Tb.Th. (G,H) Representative histological images of the distal femur in each group, including H&E and TRAP staining. Scale bars, 400 µm (G, upper row) and 100 µm (G, lower row; H). (I,J) Quantitative analysis of N.Oc/BS and Oc.S/BS on bone sections. (K,L) Quantitative analysis of the serum bone resorption biomarkers TRAP‐5b and β‐CTx. For panels B–L, n = 6 mice per group, with each mouse representing one biological replicate. Data are presented as the mean ± S.D and p values were determined using one‐way ANOVA followed by Tukey's multiple‐comparisons test. ns, not significant; ^*^ *p* < 0.05; ^**^ *p* < 0.01.

The histological analyses were consistent with the micro‐CT findings. H&E staining revealed marked thinning and rarefaction of the trabecular bone in OVX femurs, which was partially ameliorated by Clo and Asp8‐PMK‐MCs, and most prominently improved in the Clo/Asp8‐PMK‐MC group (Figure 7G). TRAP staining demonstrated a pronounced increase in the OC number and surface area in OVX mice, whereas both Clo and Asp8‐PMK‐MCs reduced OC abundance, with Clo/Asp8‐PMK‐MCs producing the greatest reduction in Oc.S/BS (Figure 7H–J). Consistent with these local histological changes, serum levels of the bone resorption markers TRAP‐5b and β‐CTx were elevated in OVX mice and decreased following Clo treatment. Further suppression was observed in the Clo/Asp8‐PMK‐MC group, and Asp8‐PMK‐MCs alone also elicited a modest reduction (Figure 7K,L). Collectively, these results indicate that Asp8‐PMK‐MCs alone reduced bone resorption‐related parameters in vivo, whereas Clo/Asp8‐PMK‐MCs produced more pronounced reductions.

To evaluate systemic safety, major organs were examined using H&E staining. No obvious histopathological abnormalities were detected in the heart, liver, spleen, lungs, or kidneys across all treatment groups, including the Clo/Asp8‐PMK‐MC group (Figure S22). This indicated that the nanocarrier formulation did not induce overt organ toxicity at any of the tested doses.

To further evaluate the systemic safety of repeated administration, an additional biosafety study was performed after six weeks of repeated intravenous injection. No notable abnormalities were observed in routine blood or serum biochemical parameters among the control, free Clo, Asp8‐PMK‐MC, and Clo/Asp8‐PMK‐MC groups (Tables S7 and S8). Moreover, H&E staining revealed no obvious histopathological changes in the major organs after repeated dosing (Figure S23). This indicated good systemic biocompatibility of the nanoplatform.

### 3.14 Clo/Asp8‐PMK‐MCs Enhance Bone Formation and Modulate PGC1β and RUNX2 Expression

Von Kossa staining of undecalcified femoral sections revealed a marked reduction in mineralized trabecular bone in OVX mice compared with that in Sham controls. Treatment with Clo, Asp8‐PMK‐MCs, or Clo/Asp8‐PMK‐MCs partially prevented OVX‐induced loss of mineralized trabecular bone, with the largest mineralized area observed in the Clo/Asp8‐PMK‐MC group (Figure 8A). However, considering that free Clo showed limited effects on osteoblast differentiation, this enhanced mineralization is likely associated mainly with the osteogenic activity of the carrier component rather than a direct anabolic effect of Clo.

**FIGURE 8:**
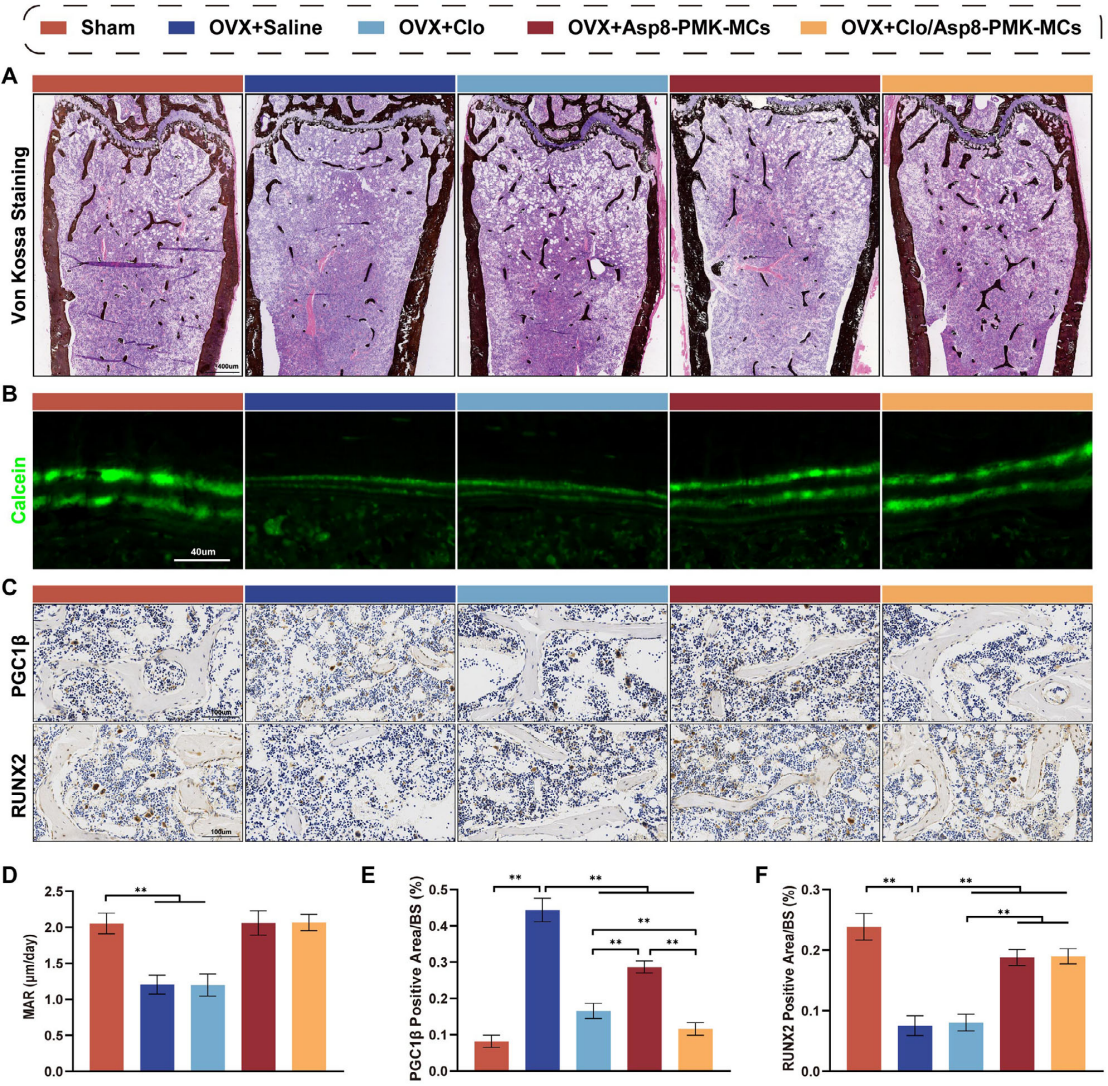
Clo/Asp8‐PMK‐MCs enhance bone formation and modulate PGC1β and RUNX2 expression. (A) Von Kossa staining of undecalcified bone sections from the Sham, OVX, Clo, Asp8‐PMK‐MCs, and Clo/Asp8‐PMK‐MCs groups. (B, D) Calcein double‐labeling performed 7 and 3 days before sacrifice to assess the mineral apposition rate (MAR). Calcein fluorescence is shown in green. (C, E, F) Immunohistochemical staining for PGC1β and RUNX2 and corresponding quantitative analysis. Scale bars, 400 µm (A), 40 µm (B), and 100 µm (C). For panels A–F, n = 6 mice per group, with each mouse representing one biological replicate. Data are presented as the mean ± S.D and p values were determined using one‐way ANOVA followed by Tukey's multiple‐comparisons test. ns, not significant; ^*^ *p* < 0.05; ^**^ *p* < 0.01.

Calcein double labeling further showed that OVX markedly reduced the MAR relative to that of the sham mice (Figure 8B,D). Clo treatment alone did not substantially improve MAR compared with that of the OVX controls. In contrast, Asp8‐PMK‐MCs clearly increased the MAR, thus indicating a pro‐osteogenic effect of the carrier. Clo/Asp8‐PMK‐MCs also elevated the MAR relative to the OVX‐ and Clo‐treated groups; however, this increase did not exceed the effect achieved by Asp8‐PMK‐MCs alone (Figure 8B,D). These findings further suggest that the enhanced osteogenic response of Clo/Asp8‐PMK‐MCs is primarily contributed by the carrier system, while Clo mainly provides anti‐resorptive activity.

Immunohistochemical analysis revealed that OVX increased PGC1β expression while reducing RUNX2 levels in bone tissue compared with that of Sham controls (Figure 8C,E,F). Both Clo and Asp8‐PMK‐MCs markedly decreased PGC1β expression, with Clo exerting a slightly stronger effect. Notably, the most pronounced suppression of PGC1β was observed in the Clo/Asp8‐PMK‐MCs group. In contrast, Clo alone had a minimal impact on RUNX2 expression, whereas treatment with Asp8‐PMK‐MCs and Clo/Asp8‐PMK‐MCs markedly increased RUNX2 expression in bone tissue (Figure 8C,E,F). To further examine the cellular localization of PGC1β in vivo, dual immunofluorescence staining for PGC1β and the osteoclast marker CTSK was performed. The merged images and enlarged views showed overlapping PGC1β and CTSK signals along the trabecular bone surface, supporting OC‐associated PGC1β localization in vivo (Figure S24). Collectively, these results indicate that although Clo alone does not enhance mineral apposition, Asp8‐PMK‐MCs promote bone formation, and their combination with Clo leads to the most robust reduction of PGC1β alongside improved expression of osteogenic markers in OVX mice.

Notably, Clo and Asp8‐PMK‐MCs exhibited distinct biological activities. Specifically, Clo primarily inhibited OC differentiation and bone resorption, with minimal effects on osteogenesis. In contrast, the blank carrier exerted modest anti‐osteoclastogenic activity, together with a more pronounced pro‐osteogenic effect. Therefore, the therapeutic effects of Clo/Asp8‐PMK‐MCs may reflect contributions from both Clo‐mediated inhibition of bone resorption and carrier‐mediated regulation of bone formation. However, as no formal synergy analysis was performed in the current study, the data did not allow for determination of additive or synergistic combined effects. Hence, this aspect warrants further investigation in future studies.

Current clinically established osteoporosis treatments, including alendronate and denosumab, primarily suppress OC‐mediated bone resorption [52]. By contrast, the present ROS‐responsive, bone‐targeted nanoplatform was designed to integrate bone‐targeted delivery, ROS‐responsive drug release, and modulation of both bone resorption and bone formation within a single formulation. Specifically, Clo predominantly inhibits osteoclastogenesis and bone resorption, whereas the Asp8‐PMK‐MCs carrier promotes osteogenic differentiation while exerting a modest anti‐osteoclastogenic effect. These distinct effects may collectively contribute to the observed therapeutic outcome, although whether their interaction is additive or synergistic remains to be determined. In addition, repurposing Clo, a clinically used antitussive drug, may provide a potential advantage for further translational development. Nevertheless, because the present study did not include direct comparisons with approved osteoporosis therapies, no conclusion can yet be drawn regarding superiority in efficacy, safety, or systemic exposure. Head‐to‐head comparative studies and further preclinical evaluation are therefore required to establish the potential clinical value of this nanoplatform.

This study had some limitations. First, molecular docking, CETSA, and functional rescue experiments suggest that PGC1β contributes to Clo action, but direct biophysical evidence of Clo–PGC1β binding remains limited. Second, although Clo reduced complex I activity, whether this reflects a primary effect on the respiratory chain or secondary mitochondrial changes remains to be clarified. Third, fluorescence tracking of DID‐labeled micelles does not directly quantify Clo, and comparative Clo concentrations in bone, plasma, and major organs were not measured following administration of free Clo and Clo/Asp8‐PMK‐MCs. Enhanced bone exposure to Clo and reduced systemic exposure therefore remain unverified. Additionally, although preliminary experiments suggested no obvious differences between PMK‐MCs and Asp8‐PMK‐MCs, the lack of a full factorial design prevented complete separation of the contributions of Clo, the carrier system, Asp8‐mediated targeting, and ROS‐responsive release. Future studies combining direct drug quantification at multiple time points with factorial experimental designs would help establish the tissue distribution of Clo and distinguish the contributions of these components. Fourth, treatment in the early intervention OVX model began one week after ovariectomy. The findings therefore primarily demonstrate attenuation of OVX‐induced bone loss rather than efficacy in established osteoporosis. Delayed‐treatment OVX studies, with bone loss established and confirmed before treatment, are needed to further evaluate Clo/Asp8‐PMK‐MCs efficacy in osteoporosis. Fifth, bone mechanical competence was not assessed. Although Clo/Asp8‐PMK‐MCs improved bone mass, microarchitecture, histological outcomes, and mineral apposition, whether these changes translate into improved mechanical performance remains unknown. Finally, because of the limited solubility of the polymers in the available GPC mobile phase, their molecular‐weight distributions and dispersities could not be determined. Future studies using optimized solvent systems or alternative methods will be required for more comprehensive molecular‐weight characterization. Further studies addressing these limitations are needed to clarify the mechanisms of action of Clo/Asp8‐PMK‐MCs and assess their translational potential for attenuating estrogen deficiency‐induced bone loss.

## 4 Conclusion

This study demonstrated that OXPHOS is progressively upregulated during OC differentiation and represents a tractable metabolic target for antiresorptive interventions. Through a literature‐guided drug repurposing screen, Clo was identified as a potent inhibitor of RANKL‐induced osteoclastogenesis by suppressing OC OXPHOS to attenuate OC differentiation and bone‐resorptive activity in vitro and markedly attenuate bone loss in ovariectomized mice. Moreover, ROS‐responsive Clo/Asp8‐PMK‐MCs combined Clo‐mediated antiresorptive activity with carrier‐mediated promotion of OB differentiation, thereby attenuating OVX‐induced bone loss and preserving trabecular microarchitecture (Figure 9). Collectively, these findings support the therapeutic potential of an OXPHOS‐targeted nanomedicine combining repurposed Clo and a bioactive carrier for attenuating estrogen deficiency‐induced bone loss.

**FIGURE 9:**
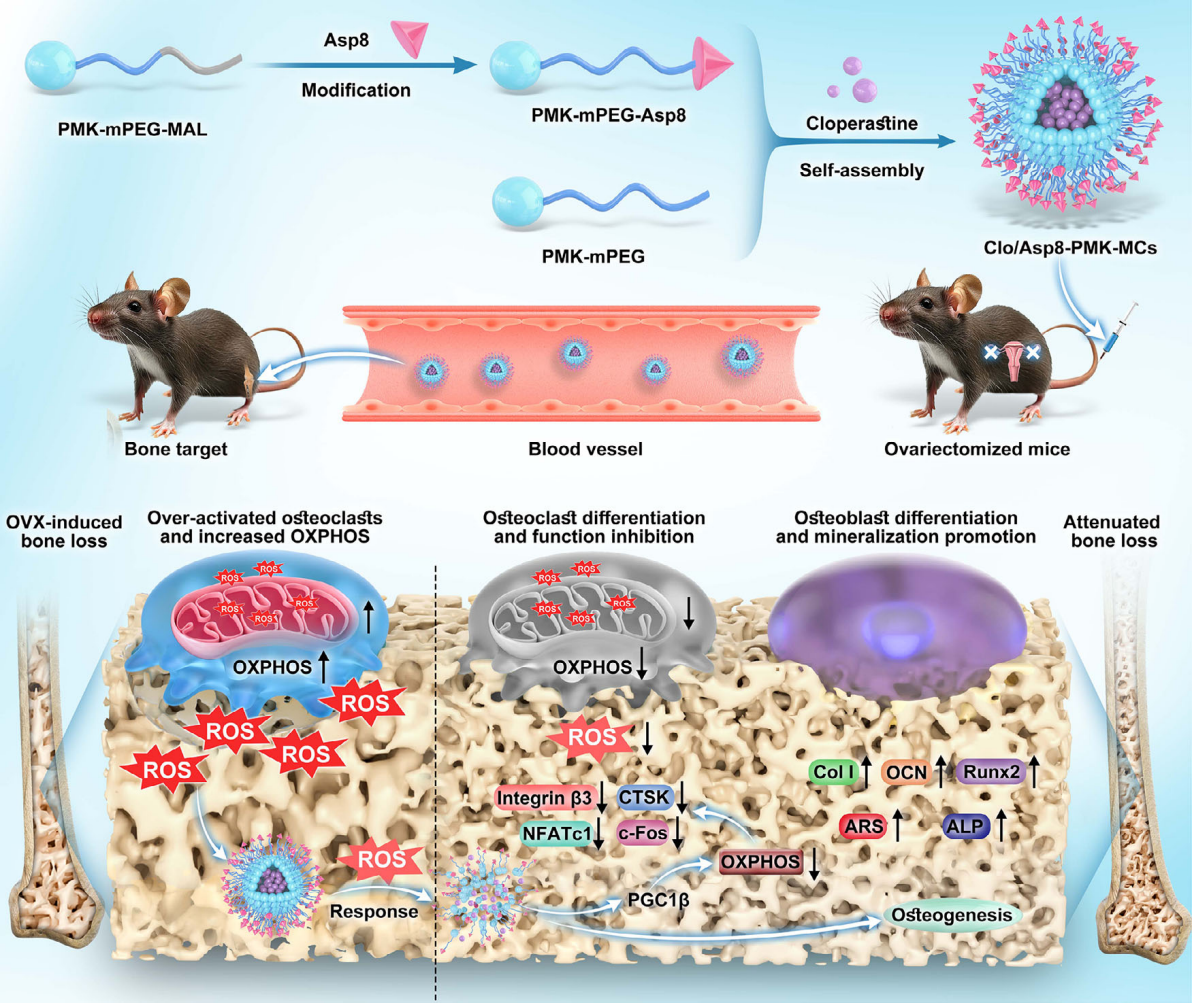
Schematic illustration of the proposed mechanisms by which Clo/Asp8‐PMK‐MCs attenuate OVX‐induced bone loss.

## Author Contributions

H.M.J., X.Y. designed the study. H.B.L., Y.X., J.T.L., Y.Y.Y., Z.Z.H and X.Q.S. performed the experiments and acquired the data. Q.H.W.,C. X. Y., J.S.M., Y.T.T., Q.C.L and B.R.Q. analyzed and interpreted the data. H.B.L. and Y.X. wrote the manuscript. H.M.J., S.C.C., X.Y.W. and J.K.X. supervised the research, managed resources, and contributed to manuscript review and editing.

## Funding

This work was supported by the National Key R&D Program of China (2024YFA0919200, 2025YFC2428200), National Natural Science Foundation of China (82202757, 52473126, 82372461, 32301097), Shenzhen Medical Research Fund (B2302005).

## Conflicts of Interest

The authors declare no conflicts of interest.

## Supporting information




**Supporting File 1**: advs78205‐sup‐0001‐SuppMat.docx.

**Supporting File 2**: advs78205‐sup‐0002‐S9 ‐clean.xlsx.

## Data Availability

The data that support the findings of this study are available from the corresponding author upon reasonable request. The RNA‐seq data generated in this study have been deposited in the Sequence Read Archive under BioProject accession number PRJNA1531789.
