# Supplementary material for: A Novel Reactive Oxygen Species‐Responsive Bone‐Targeted Nanoplatform Based on Cloperastine Attenuates Ovariectomy‐Induced Bone Loss by Inhibiting Osteoclast Oxidative Phosphorylation
Source: Adv Sci (Weinh). 2026 Oct 11:e78205. Online ahead of print. doi: 10.1002/advs.78205 (PMC13655263; doi:10.1002/advs.78205)
Supplement: Supplementary file 1 [file ADVS-9999-e78205-s002.docx]

Supporting Information

**A Novel Reactive Oxygen Species-Responsive Bone-Targeted Nanoplatform Based on Cloperastine Attenuates Ovariectomy-Induced Bone Loss by Inhibiting Osteoclast Oxidative Phosphorylation**

*Haibo Liang^1, 2#^, Yong Xiao^1, 2#^, Jiangtao Luo^1, 2#^, Xiao Yang^3#^,* *Yayun Yang^3^, Qihang Wu^1, 4^, Chenxin Yu^2, 5^, Zhihao Zhou^3^, Xiaoqing Si^3^, Jiansen Miao^1, 2^, Yiting Tu^1, 2^, Qichen Lu^1, 2^, Boreng Qian^1, 2^, Xiangyang Wang^1, 2*^, Jiake Xu^1, 6, 7^*, Changcan Shi^3^*, Haiming Jin^1, 2, 7*^*

1. Department of Orthopaedics, Key Laboratory of Orthopaedics of Zhejiang Province, The Second Affiliated Hospital and Yuying Children's Hospital of Wenzhou Medical University, Wenzhou, China;

2. School of the Second Clinical Medical Sciences, Wenzhou Medical University, Wenzhou, China

3. Wenzhou Institute, University of Chinese Academy of Sciences, Wenzhou, China;

4. Department of Spinal Surgery, Shanghai East Hospital, School of Medicine, Tongji University, Shanghai, China;

5. Department of Endocrinology, The Second Affiliated Hospital and Yuying Children's Hospital of Wenzhou Medical University, Wenzhou, China.

6. Faculty of Pharmaceutical Sciences, Shenzhen University of Advanced Technology, Shenzhen, China;

7. School of Biomedical Sciences, The University of Western Australia, Perth, Australia.

**Figure S1. Representative images from the preliminary screening of 43 candidate drugs.** Scale bar, 100μm.

**Figure S2. Secondary functional screening of the 6 prioritized OXPHOS-related compounds.** (A) Cell viability of BMMs treated with the six candidate compounds at the indicated concentrations. (B, C) Seahorse XF Mito Stress Test showing OCR curves and quantitative analysis of basal respiration, maximal respiration, mitochondrial ATP production, and spare respiratory capacity. (D, E) ECAR curves and quantitative analysis of basal ECAR and maximal ECAR. (F, G) Representative JC-1 fluorescence images and quantitative analysis of the red/green fluorescence intensity ratio. JC-1 aggregates and monomers are shown in red and green, respectively. Scale bars, 20 μm (F). All experiments used three biological replicates, each derived from BMMs independently isolated from a different mouse. Data are presented as the mean ± S.D and p values were determined using one-way ANOVA followed by Tukey’s multiple-comparisons test. ns, not significant; *p < 0.05; **p < 0.01.

**Figure S3. Effects of Clo on LDH release from BMMs**. BMMs were treated with the indicated concentrations of Clo (0–20 μM) for (A) 48 h and (B) 96 h, and cytotoxicity was evaluated by measuring LDH release into the culture supernatant. All experiments used three biological replicates per group, each derived from BMMs independently isolated from a different mouse. Data are presented as the mean ± S.D and p values were determined using one-way ANOVA followed by Tukey’s multiple-comparisons test. ns, not significant; *p < 0.05; **p < 0.01.

**Figure S4. Clo does not significantly affect glycolysis in OCs.** (A) GSEA results indicating that Clo has no obvious effect on glycolysis in OCs. (B) Representative western blots and corresponding quantification of HK2, PKM2, and LDHA in RANKL-induced OCs treated with Clo (0, 5, and 10 μM) (n = 3 biological replicates from independently isolated BMMs of three different mice). Data are presented as the mean ± S.D and p values were determined using one-way ANOVA followed by Tukey’s multiple-comparisons test. ns, not significant; *p < 0.05; **p < 0.01.

**Figure S5. Clo reduces mitochondrial respiration in BMMs**. (A) Representative Seahorse OCR traces of BMMs without RANKL after Clo treatment. (B) Quantification of basal respiration, ATP production, maximal respiration, and spare respiratory capacity. All experiments used three biological replicates per group, each derived from BMMs independently isolated from a different mouse. Data are presented as the mean ± S.D and p values were determined using unpaired two-tailed Student's t-test. ns, not significant; *p < 0.05; **p < 0.01.

**Figure S6. Clo reduces mitochondrial complex I activity in BMMs cultured with or without RANKL**. BMMs were cultured in the presence or absence of RANKL and treated with vehicle or Clo (10 μM) for 5 days (n = 3 biological replicates from independently isolated BMMs of three different mice). Data are presented as the mean ± S.D and p values were determined using one-way ANOVA followed by Tukey’s multiple-comparisons test. ns, not significant; *p < 0.05; **p < 0.01.

**Figure S7. Clo attenuates OVX-induced bone loss.** (A) Representative CT reconstruction images of the Sham, OVX, Clo (5 mg/kg), and Clo (10 mg/kg) groups. (B–E) Analysis of bone microarchitecture parameters in each group. (F) H&E staining of the distal femur. (G–I) TRAP staining of bone sections and quantitative analysis of N.Oc/BS and Oc.S/BS. Scale bars, 400 μm (F, upper row) and 100 μm (F, lower row; G). For panels A–I, n = 6 mice per group, with each mouse representing one biological replicate. Data are presented as the mean ± S.D and p values were determined using one-way ANOVA followed by Tukey’s multiple-comparisons test. ns, not significant; *p < 0.05; **p < 0.01.

**Figure S8. Structures and ¹H NMR spectra of mPEG_5K_-PMK (A, D), MAL-mPEG_5K_-PMK (B, E), and Asp8-mPEG_5K_-PMK (C, F).**

**Figure S9. Size stability of Clo/Asp8-PMK-MCs in ddH_2_O, PBS, and serum conditions** (n = 3 independently prepared formulation batches). Data are presented as the mean ± S.D and p values were determined using two-way ANOVA followed by Šídák’s multiple-comparisons test. ns, not significant; *p < 0.05; **p < 0.01.

**Figure S10. Representative DHE staining images and quantitative analysis of ROS levels in femoral bone sections from Sham and OVX mice.** (A) Representative images showing DHE fluorescence (red) and nuclear counterstaining (blue). Scale bar, 200 μm. (B) Quantitative analysis of DHE fluorescence intensity. n = 6 mice per group, with each mouse representing one biological replicate. Data are presented as the mean ± S.D and p values were determined using unpaired two-tailed Student's t-test. ns, not significant; *p < 0.05; **p < 0.01.

**Figure S11. Effects of Clo/Asp8-PMK-MCs on cytotoxicity in OCs.** (A) CCK-8 assay to evaluate the cytotoxicity of Clo/Asp8-PMK-MCs at different concentrations of Clo. (B, C) Quantitative analysis of the live-cell ratio (B) and representative Calcein AM/PI staining images (C) of cells treated with Clo, Asp8-PMK-MCs, and Clo/Asp8-PMK-MCs. Calcein AM-positive live cells are shown in green, and PI-positive dead cells are shown in red. Scale bars, 200 μm (C). For panels A–C, n = 3 biological replicates, each derived from BMMs independently isolated from a different mouse. Data are presented as the mean ± S.D and p values were determined using one-way ANOVA followed by Tukey’s multiple-comparisons test. ns, not significant; *p < 0.05; **p < 0.01.

**Figure S12. *In vivo* fluorescence tracking and *in vitro* uptake of DID/Asp8-PMK-MCs.** (A) In vivo imaging of DID/Asp8-PMK-MCs and DID/PMK-MCs (n = 3 mice per group). (B, C) Cellular uptake of DID/Asp8-PMK-MCs in BMMs and OBs at 1 h, 3 h, and 6 h (n = 3 biological replicates per cell type, each derived from a different mouse). DID fluorescence is shown in red, F-actin in green, and DAPI-stained nuclei in blue. Scale bars, 20 μm (B, C).

**Figure S13. PMK-MCs and Asp8-PMK-MCs show no significant difference in regulating osteoclastogenesis and osteogenesis.** (A, B) TRAP staining and quantitative analysis of OCs formation. (C, D) ALP staining and quantitative analysis of early osteogenic differentiation. (E, F) ARS staining and quantitative analysis of matrix mineralization. Scale bars, 1000 μm (A, C, E). For panels A–F, n = 3 biological replicates, each derived from BMMs (A, B) or primary osteoblasts (C–F) independently isolated from a different mouse. Data are presented as the mean ± S.D and p values were determined using one-way ANOVA followed by Tukey’s multiple-comparisons test. ns, not significant; *p < 0.05; **p < 0.01.

**Figure S14. Clo/Asp8-PMK-MCs inhibit the expression of osteoclast-specific genes** (n = 3 biological replicates, each derived from BMMs independently isolated from a different mouse). Data are presented as the mean ± S.D and p values were determined using one-way ANOVA followed by Tukey’s multiple-comparisons test. ns, not significant; *p < 0.05; **p < 0.01.

**Figure S15. Effects of Clo/Asp8-PMK-MCs on F-actin belt formation and function in OCs.** (A–C) Effects of Clo/Asp8-PMK-MCs on F-actin belt formation in OCs and quantitative analysis. F-actin is shown in red, and DAPI-stained nuclei are shown in blue. (D, E) Effects of Clo/Asp8-PMK-MCs on intracellular ROS levels in OCs and quantitative analysis. Fluorescence following DCFH-DA staining is shown in green, with corresponding bright-field overlays shown below. Scale bars, 200 μm (A) and 20 μm (D). For panels A–E, n = 3 biological replicates, each derived from BMMs independently isolated from a different mouse. Data are presented as the mean ± S.D and p values were determined using one-way ANOVA followed by Tukey’s multiple-comparisons test. ns, not significant; *p < 0.05; **p < 0.01.

**Figure S16. Expression pattern of PGC1β across bone-related cell lineages based on Human Protein Atlas database.**

**Figure S17. PGC1β is a potential molecular target of Clo.** (A) Changes in the expression levels of Ppargc1a and Ppargc1b in the RNA-seq data. (B) Predicted binding mode of Clo based on the PPAR–PGC1β complex structure (PDB ID: 3SP6). (C) Non-bonding interactions between PGC1β and Clo. (D) Hydrogen bonds formed between Clo and adjacent amino acid residues within PGC1β. (E, F) Western blot analysis of the effect of Clo on PGC1β under RANKL stimulation. (G, H) CETSA analysis of the effect of Clo on the thermal stability of PGC1β. (I, J) Western blot analysis of the effects of Clo, Asp8-PMK-MCs, and Clo/Asp8-PMK-MCs on PGC1β expression. For panels A and E–J, n = 3 biological replicates, each derived from BMMs independently isolated from a different mouse. Data are presented as the mean ± S.D and p values were determined using two-way ANOVA followed by Šídák’s multiple-comparisons test (F, H), one-way ANOVA followed by Tukey’s multiple-comparisons test (J). ns, not significant; *p < 0.05; **p < 0.01.

**Figure S18. Clo regulates OCs differentiation in a PGC-1β-dependent manner.** (A) RT-qPCR analysis showing the overexpression efficiency of PGC-1β mRNA following Lv-PGC-1β transduction. (B, C) Representative Western blot images and quantitative analysis confirming PGC-1β protein overexpression. (D, E) Representative Western blot images and quantitative analysis showing the protein expression of PGC-1β following Lv-PGC-1β transduction and Clo treatment. (F, G) Representative TRAP staining images and quantitative analysis demonstrating that PGC-1β overexpression partially rescued the inhibitory effect of Clo on OCs differentiation. Scale bar, 1000 μm. For panels A–G, n = 3 biological replicates, each derived from BMMs independently isolated from a different mouse. Data are presented as the mean ± S.D and p values were determined using one-way ANOVA followed by Tukey’s multiple-comparisons test. ns, not significant; *p < 0.05; **p < 0.01.

**Figure S19. Effects of PGC1β knockdown and Clo treatment on RANKL-induced OC formation and mitochondrial respiration.** (A) Relative PGC1β mRNA expression following transfection with negative control siRNA (si-NC) or three PGC1β-targeting siRNAs (si-1, si-2, and si-3). (B, C) Representative western blots and quantification of PGC1β protein expression in the indicated groups. (D, E) Representative western blots and quantification of PGC1β protein expression under RANKL stimulation following PGC1β knockdown and/or Clo treatment. (F, G) Representative TRAP staining images and quantification of OCs per well in the indicated groups. Scale bar, 1,000 μm. (H) OCR profiles following sequential addition of oligomycin, FCCP, and Rot/AA, normalized to protein content. (I) Quantification of basal respiration, maximal respiration, ATP-linked respiration, and spare respiratory capacity. For panels A–I, n = 3 biological replicates, each derived from BMMs independently isolated from a different mouse. Data are presented as the mean ± S.D and p values were determined using one-way ANOVA followed by Tukey’s multiple-comparisons test. ns, not significant; *p < 0.05; **p < 0.01.

**Figure S20. Effects of Clo and Clo/Asp8-PMK-MCs on cytotoxicity in OBs.** (A) CCK-8 assay to assess the cytotoxicity of Clo at different concentrations in OBs. (B) CCK-8 assay to evaluate the cytotoxicity of Clo/Asp8-PMK-MCs on OBs at different Clo concentrations. (C) Calcein AM/PI staining of OBs treated with Clo, Asp8-PMK-MCs, and Clo/Asp8-PMK-MCs. Calcein AM-positive live cells are shown in green, and PI-positive dead cells are shown in red. Scale bars, 200 μm (C). For panels A–C, n = 6 biological replicates, each derived from primary osteoblasts independently isolated from a different mouse. Data are presented as the mean ± S.D and p values were determined using one-way ANOVA followed by Tukey’s multiple-comparisons test. ns, not significant; *p < 0.05; **p < 0.01.

**Figure S21. Effects of Clo/Asp8-PMK-MCs on OB differentiation and mineralization.** (A, B) Effects of Clo, Asp8-PMK-MCs, and Clo/Asp8-PMK-MCs on ALP staining in OBs after 7 days of osteogenic induction and quantitative analysis. (C, D) Effects of Clo, Asp8-PMK-MCs, and Clo/Asp8-PMK-MCs on ARS staining in OBs after 21 days of osteogenic induction and quantitative analysis. Scale bars, 1000 μm (A, C). (E-H) Representative western blots and corresponding quantification of COL1A1, RUNX2, and OCN protein expression in OBs under indicated treatments. Biological replicates consisted of primary osteoblasts independently isolated from different mice (n = 6 for panels A–D; n = 3 for panels E–H). Data are presented as the mean ± S.D and p values were determined using one-way ANOVA followed by Tukey’s multiple-comparisons test. ns, not significant; *p < 0.05; **p < 0.01.

**Figure S22. H&E staining of major organs in OVX mice after treatment.** Representative H&E images of the heart, liver, spleen, lung, and kidney from the Sham, OVX, Clo, Asp8-PMK-MCs, and Clo/Asp8-PMK-MCs groups (n = 6 mice per group, with each mouse representing one biological replicate). Scale bar, 100 μm.

**Figure S23. H&E staining of major organs after 6 weeks of repeated administration.** Representative H&E images of the heart, liver, spleen, lung, and kidney from the Control, free Clo, Asp8-PMK-MCs, and Clo/Asp8-PMK-MCs groups (n = 6 mice per group, with each mouse representing one biological replicate). Scale bar, 100 μm.

**Figure S24. Dual immunofluorescence staining of PGC1β and CTSK in bone sections.** Representative images from the Sham, OVX + Saline, OVX + Clo, OVX + Asp8-PMK-MCs, and OVX + Clo/Asp8-PMK-MCs groups. PGC1β and CTSK are shown in green and red, respectively, and nuclei are counterstained with DAPI (blue). White dashed lines in the DAPI panels indicate approximate trabecular bone contours. Red boxes in the merged images indicate the regions enlarged in the bottom row. Red arrows highlight regions of overlapping PGC1β and CTSK signals along the trabecular bone surface. Scale bars: 100 μm for overview images and 20 μm for enlarged images. n = 3 mice per group, with each mouse representing one biological replicate.

Table S1. List of abbreviations.

| Abbreviation | Full term |
| --- | --- |
| OXPHOS | oxidative phosphorylation |
| Clo | cloperastine |
| RANKL | receptor activator of nuclear factor κB ligand |
| ROS | reactive oxygen species |
| ATP | adenosine triphosphate |
| M-CSF | macrophage colony-stimulating factor |
| PFA | paraformaldehyde |
| PBS | phosphate-buffered saline |
| EDTA | ethylenediaminetetraacetic acid |
| TBST | tris-buffered saline with Tween |
| DMSO | dimethyl sulfoxide |
| FBS | fetal bovine serum |
| CTSK | Cathepsin K |
| NFATc1 | Nuclear factor of activated T cells 1 |
| ATP5A | ATP synthase F1 subunit alpha |
| UQCRC2 | Ubiquinol-cytochrome c reductase core protein 2 |
| MTCO2 | Mitochondrially encoded cytochrome c oxidase II |
| SDHB | Succinate dehydrogenase complex iron sulfur subunit B |
| NDUFB8 | NADH:ubiquinone oxidoreductase subunit B8 |
| DRP1 | Dynamin-related protein 1 |
| MFN2 | Mitofusin 2 |
| OPA1 | Optic atrophy protein 1 |
| PGC-1β | Peroxisome proliferator-activated receptor gamma coactivator 1-beta |
| HK2 | Hexokinase 2 |
| PKM2 | Pyruvate kinase M2 |
| LDHA | Lactate dehydrogenase A |
| COL1A1 | Collagen type I alpha 1 chain |
| RUNX2 | Runt-related transcription factor 2 |
| OCN | Osteocalcin |
| DMF | N, N-Dimethylformamide |
| THF | Tetrahydrofuran |
| OCR | oxygen consumption rate |
| BMMs | Bone marrow–derived macrophages |
| P/S | penicillin–streptomycin |
| RIPA | radioimmunoprecipitation assay |
| TRAP | Tartrate resistant acid phosphatase |
| IF | Immunofluorescence |
| DCFH-DA | 2′,7′-dichlorodihydrofluorescein diacetate |
| ΔΨm | Mitochondrial membrane potential |
| PMK | poly (methionin-block-Nε-trifluoroacetyl-L-lysine) |
| MCs | micelles |
| micro-CT | micro-computed tomography |
| DHE | dihydroethidium |
| DLS | dynamic light scattering |
| SEM | scanning electron microscopy |
| PI | propidium iodide |
| OB | osteoblast |
| CETSA | Cellular thermal shift assay |
| ALP | Alkaline phosphatase |
| ARS | Alizarin Red staining |
| OVX | ovariectomy |
| MAR | mineral apposition rate |
| H&E | hematoxylin–eosin |
| TRACP-5b | tartrate-resistant acid phosphatase 5b |
| β-CTX | β-C-telopeptide of type I collagen |
| Mono | monocyte |
| pre-OC | pre-osteoclast |
| OC | osteoclast |
| PBMC | peripheral blood mononuclear cell |
| PCA | Principal component analysis |
| WBC | White blood cell count |
| NE | Neutrophil count |
| LY | Lymphocyte count |
| MO | Monocyte count |
| EO | Eosinophil count |
| BA | Basophil count |
| NE% | Neutrophil percentage |
| LY% | Lymphocyte percentage |
| MO% | Monocyte percentage |
| EO% | Eosinophil percentage |
| BA% | Basophil percentage |
| RBC | Red blood cell count |
| HGB | Hemoglobin |
| HCT | Hematocrit |
| MCV | Mean corpuscular volume |
| MCH | Mean corpuscular hemoglobin |
| MCHC | Mean corpuscular hemoglobin concentration |
| RDW | Red blood cell distribution width |
| PLT | Platelet count |
| MPV | Mean platelet volume |
| ALT | Alanine aminotransferase |
| AST | Aspartate aminotransferase |
| BUN | Blood urea nitrogen |
| CREA | Creatinine |
| TP | Total protein |
| ALB | Albumin |
| GLB | Globulin |
| UA | Uric acid |
| TC | Total cholesterol |
| TG | Triglycerides |
| GLU | Glucose |
| LDH | lactate dehydrogenase |

Table S2. Primary antibodies used in this study.

| Antibodies | Source | Concentration for WB | Concentration for IF | Identifier |
| --- | --- | --- | --- | --- |
| CTSK | Santa Cruz | 1:250 | 1:50 | cat# sc-48353 |
| NFATc1 | Santa Cruz | 1:250 | 1:50 | cat# sc-7294 |
| c-Fos | Abcam | 1:1000 | / | cat# ab222699 |
| ATP5A | Santa Cruz | 1:500 | / | cat# sc-136178 |
| UQCRC2 | Santa Cruz | 1:500 | / | cat# sc-390378 |
| MTCO2 | Proteintech | 1:2000 | / | cat# 55070-1-AP |
| SDHB | Proteintech | 1:2000 | / | cat# 10620-1-AP |
| NDUFB8 | Proteintech | 1:1000 | / | cat# 14794-1-AP |
| β-actin | Proteintech | 1:10000 | / | cat# 66009-1-Ig |
| DRP1 | Proteintech | 1:2000 | / | cat# 12957-1-AP |
| MFN2 | Proteintech | 1:2000 | / | cat# 12186-1-AP |
| OPA1 | Proteintech | 1:2000 | / | cat# 27733-1-AP |
| PGC-1β | Proteintech | 1:1000 | 1:100 | cat# 22378-1-AP |
| integrin β3 | Proteintech | 1:1000 | / | cat# 18309-1-AP |
| HK2 | Proteintech | 1:1000 | / | cat# 22029-1-AP |
| PKM2 | Proteintech | 1:2000 | / | cat# 15822-1-AP |
| LDHA | Proteintech | 1:2000 | / | cat# 19987-1-AP |
| COL1A1 | Proteintech | 1:2000 | / | cat# 66761-1-Ig |
| RUNX2 | Proteintech | 1:1000 | / | cat# 20700-1-AP |
| OCN | Proteintech | 1:1000 | / | cat# 23418-1-AP |

Table S3. Screening compounds and their literature-reported metabolic effects.

| Compound | Literature-reported effects on OXPHOS or related energy metabolism | Supporting references |
| --- | --- | --- |
| Atovaquone | Blocks mitochondrial respiratory-chain complexes I, II, and III. | Oxidative phosphorylation inhibitors inhibit proliferation of endometriosis cells |
| mubritinib | Reduces PI3K/mTOR signaling activation, disrupts mitochondrial function, markedly increases ROS levels, and induces oxidative stress. | Mubritinib enhanced the inhibiting function of cisplatin in lung cancer by interfering with mitochondrial function |
| ivosidenib | A mitochondrial inhibitor that blocks tricarboxylic acid cycle enzymes or electron transport chain complexes. | Targeting mitochondrial metabolism in acute myeloid leukemia |
| venetoclax | A mitochondrial inhibitor that blocks tricarboxylic acid cycle enzymes or electron transport chain complexes. | Targeting mitochondrial metabolism in acute myeloid leukemia |
| Enasidenib | A mitochondrial inhibitor that blocks tricarboxylic acid cycle enzymes or electron transport chain complexes. | Targeting mitochondrial metabolism in acute myeloid leukemia |
| Pyrvinium pamoate | Mediates the reverse reaction of mitochondrial electron transport chain complex II. | Reprofiling a classical anthelmintic, pyrvinium pamoate, as an anti-cancer drug targeting mitochondrial respiration |
| Tigecyl(tigecycline) | Inhibits mitochondrial gene expression. | Differential contribution of the mitochondrial translation pathway to the survival of diffuse large B-cell lymphoma subsets |
| chloramphenicol | Inhibits mitochondrial gene expression (a cytochrome P450 inhibitor that reduces ROS). | Florfenicol-induced Mitochondrial Dysfunction Suppresses Cell Proliferation and Autophagy in Fibroblasts |
| cloperastine | Inhibits mitochondrial oxidative phosphorylation. | Cloperastine inhibits esophageal squamous cell carcinoma proliferation in vivo and in vitro by suppressing mitochondrial oxidative phosphorylation |
| levodopa | Downregulates oxidative phosphorylation and is associated with mitochondrial dysfunction. | Proteomics Reveal the Inhibitory Mechanism of Levodopa Against Esophageal Squamous Cell Carcinoma |
| nitazoxanide | inhibit mitochondrial respiration | Three-Dimensional Cell Culture-Based Screening Identifies the Anthelmintic Drug Nitazoxanide as a Candidate for Treatment of Colorectal Cancer |
| niclosamide | inhibit mitochondrial respiration | Three-Dimensional Cell Culture-Based Screening Identifies the Anthelmintic Drug Nitazoxanide as a Candidate for Treatment of Colorectal Cancer |
| closantel | inhibit mitochondrial respiration | Three-Dimensional Cell Culture-Based Screening Identifies the Anthelmintic Drug Nitazoxanide as a Candidate for Treatment of Colorectal Cancer |
| pyrvinium pamoate | inhibit mitochondrial respiration | Three-Dimensional Cell Culture-Based Screening Identifies the Anthelmintic Drug Nitazoxanide as a Candidate for Treatment of Colorectal Cancer |
| salinomycin | inhibit mitochondrial respiration | Three-Dimensional Cell Culture-Based Screening Identifies the Anthelmintic Drug Nitazoxanide as a Candidate for Treatment of Colorectal Cancer |
| metformin | Metformin reduces mitochondrial respiration during HCMV infection | Inhibiting cytomegalovirus replication through targeting the host electron transport chain |
| methiothepin | Impairs mitochondrial function, induces endoplasmic reticulum stress, reduces oxidative phosphorylation, and triggers a metabolic shift in human placental choriocarcinoma cells. | Tumor-suppressive function of methiothepin in human placental choriocarcinoma cells |
| marizomib | Acts as a dual inhibitor of the proteasome and oxidative phosphorylation (OXPHOS). | Marizomib suppresses triple-negative breast cancer via proteasome and oxidative phosphorylation inhibition |
| Thiazolides | Inhibits the mitochondrial respiratory chain. | Thiazolides promote G1 cell cycle arrest in colorectal cancer cells by targeting the mitochondrial respiratory chain |
| bedaquiline | Targets the gamma subunit (ATP5F1C) of ATP synthase. | Bedaquiline, an FDA-approved drug, inhibits mitochondrial ATP production and metastasis in vivo, by targeting the gamma subunit (ATP5F1C) of the ATP synthase |
| leflunomide | Promotes mitochondrial fusion and reduces mitochondrial mass to inhibit OXPHOS. | Mitochondrial fusion exploits a therapeutic vulnerability of pancreatic cancer |
| papaverine | Inhibits mitochondrial complex I. | Papaverine and its derivatives radiosensitize solid tumors by inhibiting mitochondrial metabolism |
| Zafirlukast | Inhibits type 2 NADH dehydrogenase and aerobic respiration. | Type 2 NADH Dehydrogenase Is the Only Point of Entry for Electrons into the Streptococcus agalactiae Respiratory Chain and Is a Potential Drug Target |
| canagliflozin | Inhibits mitochondrial electron transport chain complex I. | Canagliflozin mediated dual inhibition of mitochondrial glutamate dehydrogenase and complex I: an off-target adverse effect |
| cetylpyridinium chloride | The antiestrogenic activities of cetylpyridinium chloride and benzalkonium chloride may be associated with their mitochondrial inhibitory activities. | In Vitro Evaluation of Mitochondrial Function and Estrogen Signaling in Cell Lines Exposed to the Antiseptic Cetylpyridinium Chloride |
| benzalkonium chloride | The antiestrogenic activities of cetylpyridinium chloride and benzalkonium chloride may be associated with their mitochondrial inhibitory activities. | In Vitro Evaluation of Mitochondrial Function and Estrogen Signaling in Cell Lines Exposed to the Antiseptic Cetylpyridinium Chloride |
| Sorafenib | Identified in the cited study as a potent inhibitor of mitochondrial respiration. | Coordinate β-adrenergic inhibition of mitochondrial activity and angiogenesis arrest tumor growth |
| Regorafenib | Identified in the cited study as a potent inhibitor of mitochondrial respiration. | Coordinate β-adrenergic inhibition of mitochondrial activity and angiogenesis arrest tumor growth |
| Ponatinib | Identified in the cited study as a potent inhibitor of mitochondrial respiration. | Coordinate β-adrenergic inhibition of mitochondrial activity and angiogenesis arrest tumor growth |
| Itraconazole | Identified in the cited study as a potent inhibitor of mitochondrial respiration. | Coordinate β-adrenergic inhibition of mitochondrial activity and angiogenesis arrest tumor growth |
| Telaprevir | Identified in the cited study as a potent inhibitor of mitochondrial respiration. | Coordinate β-adrenergic inhibition of mitochondrial activity and angiogenesis arrest tumor growth |
| Sulfameter | Identified in the cited study as a potent inhibitor of mitochondrial respiration. | Coordinate β-adrenergic inhibition of mitochondrial activity and angiogenesis arrest tumor growth |
| Crystal violet | Identified in the cited study as a potent inhibitor of mitochondrial respiration. | Coordinate β-adrenergic inhibition of mitochondrial activity and angiogenesis arrest tumor growth |
| Butoconazole | Identified in the cited study as a potent inhibitor of mitochondrial respiration. | Coordinate β-adrenergic inhibition of mitochondrial activity and angiogenesis arrest tumor growth |
| Nebivolol | Identified in the cited study as a potent inhibitor of mitochondrial respiration. | Coordinate β-adrenergic inhibition of mitochondrial activity and angiogenesis arrest tumor growth |
| Pazopanib | Identified in the cited study as a potent inhibitor of mitochondrial respiration. | Coordinate β-adrenergic inhibition of mitochondrial activity and angiogenesis arrest tumor growth |
| Lomerizine | Identified in the cited study as a potent inhibitor of mitochondrial respiration. | Coordinate β-adrenergic inhibition of mitochondrial activity and angiogenesis arrest tumor growth |
| Glyburide | Identified in the cited study as a potent inhibitor of mitochondrial respiration. | Coordinate β-adrenergic inhibition of mitochondrial activity and angiogenesis arrest tumor growth |
| Cimetidine | Identified in the cited study as a potent inhibitor of mitochondrial respiration. | Coordinate β-adrenergic inhibition of mitochondrial activity and angiogenesis arrest tumor growth |
| Cyclobenzaprine | Cyclobenzaprine caused on L. infantum promastigotes a decrease of intracellular ATP levels, with irreversible depolarization of plasma membrane, the collapse of the mitochondrial electrochemical potential, mild uncoupling of the respiratory chain. | Energy metabolism as a target for cyclobenzaprine: A drug candidate against Visceral Leishmaniasis |
| edaravone | Acts as an enhancer of mitochondrial metabolism. | Dysfunctional oxidative phosphorylation shunts branched‐chain amino acid catabolism onto lipogenesis in skeletal muscle |
| Dimethyl fumarate | Inhibits aerobic glycolysis and oxidative phosphorylation in tumor cells. | Taming metabolic competition via glycolysis inhibition for safe and potent tumor immunotherapy |
| clofazimine | Associated with mitochondrial dysfunction and oxidative phosphorylation. | Single-Cell Proteomics and Tumor RNAseq Identify Novel Pathways Associated With Clofazimine Sensitivity in PI- and IMiD- Resistant Myeloma, and Putative Stem-Like Cells |

Table S4. Primer sequences used for RT-qPCR.

| Gene | Forward primer (5'->3') | Reverse primer (5'->3') |
| --- | --- | --- |
| Ppargc1b | CTTGGCTGCGCTTACGAAGA | GAAAGCTCGTCCACGTCAGAC |
| NFATc1 | CTGCAACAAGCGCAAGTACA | AGGTCCAGAGTGCTATCGGT |
| CTSK | GCACCCTTAGTCTTCCGCTC | ACCCACATCCTGCTGTTGAG |
| Itgb3 | TTACCACGGATGCCAAGACC | CCCCAGAGATGGGTAGTCCA |
| Fos | TGTTCCTGGCAATAGCGTGT | TCAGACCACCTCGACAATGC |
| ACTB | GGCTGTATTCCCCTCCATCG | CCAGTTGGTAACAATGCCATGT |

| siRNA | Sequence | |
| --- | --- | --- |
|  | sense（5'-3'） | antisense（5'-3'） |
| si-1 | GUACGGAACUGCAUAAGCATT | UGCUUAUGCAGUUCCGUACTT |
| si-2 | CUGAGUUCUCUAUCCUAAGTT | CUUAGGAUAGAGAACUCAGTT |
| si-3 | CUUGUCACAAAGCGACUCUTT | AGAGUCGCUUUGUGACAAGTT |

Table S5. siRNA Sequence.

| Polymer | DP_PMK,NMR_ | Designed Met:Lys(TFA) ratio | M_n, NMR_(kDa)^a^ |
| --- | --- | --- | --- |
| mPEG-PMK | 53.0 | 18:30 | 15.0 |
| MAL-mPEG-PMK | 51.8 | 18:30 | 15.0 |

Table S6. Calculated molecular weights of the polymers.

a The total degree of polymerization of the PMK segment was estimated from the integral ratio of the polypeptide α-CH signals (δ 4.02–4.55 ppm, 1 H per residue) to the PEG backbone signals (δ 3.40–3.75 ppm, approximately 454 H for PEG_5K_). Because the Met and Lys(TFA) resonances partially overlapped, their individual degrees of polymerization could not be determined independently, and the designed Met(TFA) molar ratio of 18:30 was used for the molecular-weight estimation. The molecular-weight contribution of the maleimide-containing linker was included for MAL-PEG_5K_-PMK. These values represent ^1^H NMR-estimated number-average molecular weights. Dispersity was not determined.

Table S7. Hematological parameters of mice after repeated treatments.

| Parameter | Units | Control | Free Clo | Asp8-PMK-MCs | Clo/Asp8-PMK-MCs |
| --- | --- | --- | --- | --- | --- |
| WBC | 10^3^/μL | 4.99±1.63 | 4.95±1.69 | 5.04±1.27 | 5.01±1.05 |
| NE | 10^3^/μL | 0.68±0.30 | 0.73±0.31 | 0.69±0.22 | 0.70±0.28 |
| LY | 10^3^/μL | 4.02±1.62 | 3.95±1.47 | 4.05±1.31 | 4.01±1.11 |
| MO | 10^3^/μL | 0.21±0.052 | 0.20±0.046 | 0.22±0.072 | 0.22±0.054 |
| EO | 10^3^/μL | 0.08±0.04 | 0.07±0.03 | 0.08±0.04 | 0.08±0.02 |
| BA | 10^3^/μL | 0.00±0.00 | 0.00±0.00 | 0.00±0.00 | 0.00±0.00 |
| NE% | % | 14.70±6.92 | 14.56±4.70 | 14.42±5.31 | 14.72±6.44 |
| LY% | % | 78.83±7.69 | 79.19±4.45 | 79.34±6.40 | 79.02±7.88 |
| MO% | % | 4.69±2.19 | 4.57±2.43 | 4.62±2.04 | 4.52±1.53 |
| EO% | % | 1.78±1.01 | 1.69±1.24 | 1.62±0.70 | 1.75±0.78 |
| BA% | % | 0.00±0.00 | 0.00±0.00 | 0.00±0.00 | 0.00±0.00 |
| RBC | 10^6^/μL | 8.81±0.71 | 8.69±0.52 | 8.45±0.68 | 8.58±0.54 |
| HGB | g/dL | 11.83±1.01 | 11.61±0.77 | 11.95±0.82 | 12.00±1.13 |
| HCT | % | 39.73±2.32 | 37.63±2.27 | 38.43±4.02 | 38.38±2.75 |
| MCV | fL | 45.17±1.56 | 43.33±1.63 | 45.43±1.83 | 44.74±1.81 |
| MCH | pg | 13.50±1.60 | 13.41±1.31 | 14.22±1.48 | 14.05±1.76 |
| MCHC | g/dL | 29.87±3.24 | 30.92±2.44 | 31.40±4.04 | 31.48±4.45 |
| RDW | % | 12.73±0.48 | 12.79±0.57 | 13.16±0.57 | 12.55±0.45 |
| PLT | 10^3^/μL | 613.36±204.76 | 612.11±195.64 | 608.03±203.46 | 610.45±198.64 |
| MPV | fL | 5.27±0.70 | 5.13±0.78 | 5.19±0.77 | 5.32±0.83 |

Data are presented as the mean ± SD (n = 6 mice per group). Statistical comparisons were performed using one-way analysis of variance (ANOVA), followed by Tukey’s multiple-comparisons test. No statistically significant differences were observed among the groups (all P > 0.05).

Table S8. Serum biochemical parameters of mice after repeated treatments.

| Parameter | Units | Control | Free Clo | Asp8-PMK-MCs | Clo/Asp8-PMK-MCs |
| --- | --- | --- | --- | --- | --- |
| ALT | U/L | 38.91±3.01 | 39.13±3.40 | 38.64±4.55 | 40.18±4.36 |
| AST | U/L | 136.05±16.34 | 138.50±11.80 | 139.16±18.55 | 130.84±16.46 |
| BUN | mg/dL | 25.11±3.08 | 23.52±3.86 | 24.23±3.19 | 23.70±4.01 |
| CREA | mg/dL | 0.23±0.012 | 0.28±0.036 | 0.22±0.013 | 0.24±0.028 |
| TP | g/dL | 5.90±0.66 | 5.88±0.51 | 6.03±0.44 | 5.72±0.54 |
| ALB | g/dL | 2.98±0.36 | 2.91±0.39 | 3.07±0.34 | 2.91±0.27 |
| GLB | g/dL | 2.92±0.65 | 2.97±0.54 | 2.96±0.47 | 2.81±0.53 |
| UA | mg/dL | 1.09±0.12 | 1.05±0.10 | 1.03±0.11 | 1.00±0.13 |
| TC | mg/dL | 68.69±15.70 | 67.09±14.62 | 66.91±13.53 | 69.09±13.12 |
| TG | mg/dL | 81.19±23.87 | 79.35±25.76 | 80.27±26.28 | 81.15±26.04 |
| GLU | mg/dL | 248.38±35.94 | 249.03±36.57 | 254.21±38.13 | 252.89±36.53 |

Data are presented as the mean ± SD (n = 6 mice per group). Statistical comparisons were performed using one-way analysis of variance (ANOVA), followed by Tukey’s multiple-comparisons test. No statistically significant differences were observed among the groups (all P > 0.05).

Table S9. P values for statistical comparisons in the main and supplementary figures.

Provided as a separate Excel workbook. Each worksheet corresponds to a main or supplementary figure and lists the panel, outcome measure, comparison, and corresponding P value.
